# Vascularised organoids: Recent advances and applications in cancer research

**DOI:** 10.1002/ctm2.70258

**Published:** 2025-03-05

**Authors:** Rui Zhou, Dagmar Brislinger, Julia Fuchs, Alicia Lyons, Sonja Langthaler, Charlotte A. E. Hauser, Christian Baumgartner

**Affiliations:** ^1^ Institute of Health Care Engineering with European Testing Center of Medical Devices Graz University of Technology Graz Austria; ^2^ Department of Cell Biology Histology and Embryology Gottfried Schatz Research Center Medical University of Graz Graz Austria

**Keywords:** 3D tissue models, angiogenesis and vasculogenesis, cancer‐vasculature interactions, drug testing platforms, extracellular matrix (ECM), preclinical cancer models, tumour biology and cancer progression, tumour microenvironment, vascularised organoids

## Abstract

Organoids are three‐dimensional (3D) cellular models designed to replicate human tissues and organs while preserving their physiological complexity and functionality. Among these, vascularised organoids represent a groundbreaking advancement in 3D tissue engineering, incorporating vascular networks into engineered tissues to more accurately mimic the in vivo tumour microenvironment. These models offer significantly improved physiological relevance compared to conventional two‐dimensional cultures or animal models, positioning them as invaluable tools in cancer research. Despite their potential, the rapid proliferation of techniques and materials for developing vascularised organoids presents challenges for researchers navigating this dynamic field. This systematic review provides a comprehensive examination of methodologies for fabricating vascularised organoids, with a focus on strategies that enhance vascularisation and support organoid growth. It critically evaluates the materials used, emphasising those that effectively mimic the extracellular matrix and facilitate vascular network formation. Key advancements in engineered organoids models are highlighted, emphasising their potential for studying interactions between vasculature and cancer cells, conducting drug screening, and understanding cytokine regulation. In summary, this review provides an in‐depth overview of the current landscape of vascularised organoid fabrication and functionality, addressing challenges and opportunities within the field. A detailed understanding of the scope and future trajectories is essential for advancing organoid development and expanding their applications in both basic cancer research and clinical practice.

**Key points:**

- Comparative analysis: Evaluation of organoids, animal models, and 2D models, highlighting their respective strengths and limitations in replicating physiological conditions and studying disease processes.
- Vascularisation techniques: Comparative evaluation of vascularised organoid fabrication methods, emphasising their efficiency, scalability and ability to replicate physiological vascular networks.
- Material selection: Thorough evaluation of materials for vascularised organoid culture system, focusing on those that effectively mimic the extracellular matrix and support vascular network formation.
- Applications: Overview of organoid applications in basic cancer research and clinical settings, with an emphasis on their potential in drug discovery, disease modelling and exploring complex biological processes.

## Graphical abstract

This review explores vascularised organoids in oncology, highlighting their advantages over 2D cultures and animal models in replicating the tumour microenvironment. It examines fabrication methods, vascularisation techniques and advancements in biomaterials, emphasising their applications in studying vasculature–cancer interactions, drug screening and advancing cancer research and clinical practice.

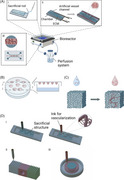

## 1 BACKGROUND

**FIGURE 1:**
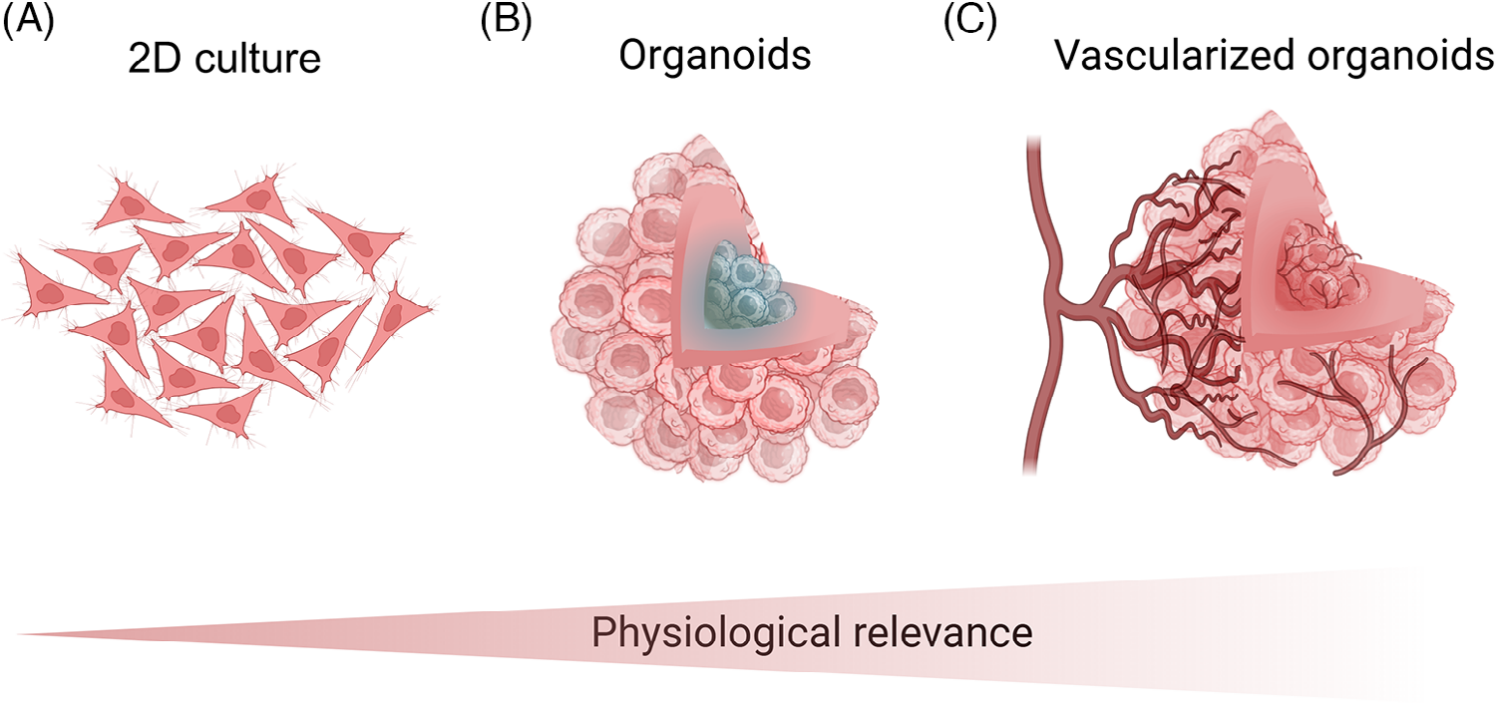
Schematic representation of different preclinical tumour models with varying levels of physiological relevance: (A) Traditional two‐dimensional monolayer cell cultures. (B) Three‐dimensional spheroid models with an apoptotic core. (C) Formation of vascularised organoids, demonstrating the integration of blood vessels within the organoid structure, which is crucial for delivering sufficient nutrients and oxygen to the central cells of the organoid. Created with BioRender.com.

Despite advancements in early diagnosis and treatment that helped lower global cancer mortality rates between 2005 and 2015,^1^ cancer remains a leading contributor to the global disease burden.^2^ For example, in 2021, lung cancer ranked as the sixth‐leading cause of death worldwide.^3^ To deepen our understanding of cancer mechanisms and improve treatment strategies, researchers have focused on developing in vitro tumour models that more accurately replicate in vivo conditions.^4^ These models serve as essential platforms for investigating tumour biology and testing potential therapies. However, traditional preclinical models, such as two‐dimensional (2D) monolayer cultures (Figure 1A) and animal models, frequently fall short in translating promising therapies into successful clinical outcomes.^5^

### 1.1 Organoids

Organoids offer a highly promising alternative for cancer research, providing physiologically relevant models that closely align with human conditions. These systems deliver genetically and phenotypically stable representations of tumours, faithfully mirroring the pathophysiological changes associated with malignancies.^6^, ^7^ By definition, organoids are in vitro self‐organising multicellular structures that exhibit cytoarchitectural and functional features with greater physiological relevance to their corresponding in vivo tissue than traditional spheroids.^8^, ^9^, ^10^ While the term ‘spheroids’ is sometimes used interchangeably, it is important to distinguish between the two. Spheroids are simpler aggregates of spherical cell clusters, characterised by lower structural complexity, limited self‐organisation and reduced biocompatibility compared to organoids.^10^, ^11^

Organoid‐based models offer numerous advantages over traditional animal and 2D models (Table 1). Animal models are often limited by high costs, ethical concerns and significant interspecies differences that can compromise the accuracy of organ and biosystem functionality.^12^ In contrast, organoids, particularly those derived from human cells, are emerging as a more physiologically relevant alternative due to their closer resemblance to human conditions and enhanced suitability for targeted biological studies.^8^, ^13^ Compared to 2D models, three‐dimensional (3D) organoid models (Figure 1B) have demonstrated a superior ability to replicate the complex architectures and mechanomimetic properties of tissues, providing a more accurate representation of the in vivo tumour microenvironment.^7^, ^14^

**TABLE 1:** Comparative analysis of 2D models, organoids and animal models.^7^, ^8^, ^11^, ^12^, ^15^

	Advantages	Limitations	Best use cases
2D models	Easy to establish and implement Widely available as commercial products	Low biocompatibility Absence of complex structures and dynamic systems	Material biocompatibility testing Cell‐level research
Organoids	Complex architectures and mechanomimetic properties Accurately replicate human tissue functions Relatively fast experimental outcomes	Lack immune system involvement Limited inter‐organ signalling	High‐throughput screening Precision medicine Organ‐level research
Animal models	Fully functional immune and circulatory systems	Time‐intensive High costs Ethical concerns Interspecies variability	Pre‐clinical testing Whole‐body research

3D organoid models in cancer research display distinct proliferation capacities, morphologies, motilities, and greater resistance to anti‐cancer drugs.^16^, ^17^ However, the tight junctions within organoids restrict oxygen penetration, leading to central cell necrosis and the development of an apoptotic core.^18^ This outcome is non‐physiological, as in vivo tissues are vascularised to facilitate sufficient oxygen and nutrient delivery, preventing such cell death. The absence of vascularisation in organoids results in hypoxic conditions and nutrient deprivation – factors not typically observed in vivo. Therefore, the formation of functional vasculature is critical for supporting long‐term survival and replicating essential biological processes in vitro, ensuring adequate nutrient and oxygen supply.^7^, ^19^

### 1.2 Vascularisation

In vivo, native blood vessel formation is driven by endothelial cells, which migrate and organise to form the inner lining of the vascular system, creating tubular structures and capillaries.^20^ As semi‐permeable membranes, endothelial cells regulate the exchange of fluids, molecules, and cells between the bloodstream and surrounding tissues.^21^ Angiogenesis, the process by which endothelial cells sprout from pre‐existing vessels, further expands and remodels the vascular network.^22^ Like non‐oncogenic tissues, tumours depend on access to the blood circulation system to acquire oxygen and nutrients essential for growth and proliferation. When pro‐angiogenic signalling dominates, triggering the so‐called ‘angiogenic switch’, the formation of new blood vessels can activate dormant tumours.^23^ However, the morphology and function of tumour‐associated vasculature differ significantly from normal blood vessels. Tumour‐associated vessels form a disorganised network characterised by reduced blood flow, excessive endothelial sprouting, inadequate pericyte coverage, disrupted endothelial junctions, increased permeability, and irregular thickness of the endothelial basement membrane.^24^

Evaluating the quality and functionality of blood vessels in organoid models typically involves the use of biomarkers. These include endothelial cell markers such as CD31 (PECAM‐1) and von Willebrand factor (vWF), as well as angiogenic factors like Vascular Endothelial Growth Factor (VEGF) and Matrix MetalloProteinases (MMPs). Additionally, analyses of vessel architecture – including diameter, branching patterns and total vascular area – along with assessments of lumen formation and vessel permeability, offer valuable insights into the functionality of blood vessels in organoid models.^25^

To accurately replicate the tumour microenvironment in vitro, the establishment of a vascularised organoid system is essential (Figure 1C). 3D vascularisation models offer critical insights into tumour growth^9^ and invasion mechanisms,^26^ while also aiding in the exploration of therapeutic strategies – particularly by revealing how the vascular network influences drug delivery.^14^ Recent advances in technologies such as lab‐on‐a‐chip (LoC) systems,^7^, ^27^ 3D bioprinting,^28^, ^29^ and extracellular matrix‐mimicking scaffolds^9^, ^17^, ^30^ have enabled the creation of vascularised organoids, ranging from tens of microns to several millimetres in diameter.^31^ However, significant challenges remain, including replicating the dynamic nature of blood flow and ensuring the proper integration and functionality of engineered blood vessels within these organoids.

### 1.3 Extracellular matrix (ECM)

The ECM is a complex network of proteins, glycoproteins and polysaccharides that provides structural and biochemical support to surrounding cell.^32^ Key components such as collagen, elastin, fibronectin and proteoglycans contribute to tissue elasticity, strength and cellular adhesion.^33^ The ECM plays a vital role in regulating cellular behaviours, including proliferation, migration, and differentiation, thereby influencing tissue development, repair and homeostasis.^34^ A major challenge in organoid development is selecting materials that effectively mimic the ECM to support cell proliferation and provide an appropriate mechanical and chemical environment. Hydrogels are widely used in organoid culture systems due to their porous structure, which enhances oxygen diffusion and nutrient supply to the dense cell mass.^35^, ^36^ Naturally derived hydrogels, like Matrigel and collagen, closely replicate the in vivo ECM microenvironment and are essential for supporting organoid metastasis.^37^ In contrast, synthetic hydrogels offer greater tunability of mechanical properties and batch‐to‐batch consistency,^38^ but often lack the bioactive signals and tissue‐like dynamic behaviour of natural hydrogels,^39^, ^40^ resulting in reduced biocompatibility and cell affinity compared to natural polymer‐based hydrogels.^41^

To address these challenges, research efforts have concentrated on two primary areas: gaining a deeper understanding of traditional materials and developing novel alternatives. Unlike non‐vascularised organoid culture systems, vascularised organoid often require additional components such as endothelial cells, fibroblasts, and specific biomolecules like VEGF to promote vasculogenesis and angiogenesis.^40^, ^42^ The ECM components and materials utilised for vascularisation play a crucial role in influencing tumour progression, cell morphology, and ultimately, the reliability of in vitro models.^12^ The diverse and complex material requirements for different aspects of the culture system present significant challenges for researchers in identifying suitable materials.

An ideal vascularised organoid model should incorporate a perfusable vascular network capable of delivering nutrients and gases uniformly throughout the organoid, functional organoid structures, an ECM‐mimicking environment, and a dynamic circulation system to support metabolism. However, the development of vascularised organoid systems is a highly complex and interdisciplinary endeavour, requiring the integration of bioengineering, materials science, and cancer cell biology principles.^43^

This review systematically explores methodologies and strategies for engineering 3D organoids with integrated vascular systems, emphasising advanced techniques for constructing functional vascular networks. It evaluates the qualitative and quantitative roles of materials in influencing organoid development, cancer cell proliferation and phenotypic expression.

To assist researchers, the review presents detailed summaries of critical information for each model in clear, organised formats such as tables and recommendations. These resources aim to simplify access to essential materials and techniques for designing or improving vascularised organoid systems. With a particular emphasis on vascularised organoids for cancer research, the review addresses knowledge gaps caused by the unique microenvironments of tumour organoids compared to healthy counterparts. The presence of cancer cells profoundly influences vascular formation and plays a pivotal role in the development of anti‐cancer therapies. These distinctions underline the importance of tailoring vascularised organoid systems for cancer‐specific studies. Finally, the review identifies the limitations in current approaches, proposes innovative solutions and highlights strategies to enhance the physiological relevance of in vitro platforms for cancer research and therapeutic development.

## 2 LITERATURE SEARCH AND CATEGORISATION

This systematic review was conducted following the guidelines of The PRISMA 2020 statement for systematic reviews.^44^ The search targeted full‐text articles published in English from 2008 to 2023. Details of the literature search strategy, study selection criteria and data extraction methods are provided in Supporting Information, section Literature selection.

The systematic literature search conducted through PubMed and Web of Science initially identified 207 potentially relevant articles. After the removal of 49 duplicate entries, a total of 158 unique articles remained. Following a thorough screening of titles and abstracts, 110 publications were excluded based on the predefined criteria. Three studies were excluded because of unavailability of full text. Full‐text evaluation of the remaining studies resulted in the exclusion of an additional 15 papers. However, reference tracking led to the inclusion of seven more relevant articles. In total, 37 studies were selected for systematic analysis (Figure S1 in Supporting Information).

This review provides a critical analysis of selected studies on vascularised organoids in cancer research, with a focus on various 3D cell culture and fabrication techniques, materials used, and their specific applications. The content is structured into two main sections: *Fabrication techniques of vascularised organoids*, and *Applications of vascularised organoids in cancer research*.

Tables 2 and 3 summarise the core findings extracted from the selected studies. Table 2 presents an overview of the methods used to construct the culture systems, the cell types employed for both cancer research and organoid culture, and the vascularisation strategies used to promote the formation of vascular networks. Table 3 provides a detailed summary of the materials used for chambers/scaffolds, ECM mimics, and vascular grafts.

**TABLE 2:** Summary of various types of vascularised 3D cell culture models including cell types and vascularisation strategies.

Lab‐on‐a‐chip			
Cells for cancer research	Cells for organoids/3D tissue models	Vascularisation strategy	Ref
MDA‐MB‐231; MCF‐7; Hs‐578T	iPSC‐ECs	Constructed artificial vascular channels	^45^
GB3 GSCs	GB3 GSCs	Constructed artificial vascular channels	^46^
Breast cancer‐associated fibroblasts	HLECs + fibroblasts; HUVEC + fibroblasts	Constructed artificial vascular channels	^47^
MDA‐MB‐231	HLECs + fibronectin	Constructed artificial vascular channels	^48^
786‐O	786‐O	Constructed artificial vascular channels	^49^
A431; BCSC	A431; BCSC	Constructed artificial vascular channels; Self‐assembled	^7^
MDA‐MB‐231‐GFP	Human pancreatic adenocarcinoma differentiated from patient stem cells	Constructed artificial vascular channels	^50^
HepG2; MDA‐MB‐231‐GFP	HepG2; MDA‐MB‐231‐GFP	Constructed artificial vascular channels	^51^
U87MG	U87MG	Constructed artificial vascular channels; Self‐assembled	^52^
hPDAC	hPDAC	Constructed artificial vascular channels	^53^
MDA‐MB‐231	MDA‐MB‐231; HUVEC	Constructed artificial vascular channels;	^54^
U87MG; HCT116; SW 480	U87MG, HCT116, and SW 480 with lung fibroblast	Self‐assembled	^55^
SKOV3; OV90; OVCAR3	SKOV3; OV90; OVCAR3	Self‐assembled	^56^
MDA‐MB‐231	NHLFs; MDA‐MB‐231	Self‐assembled	^57^
A549	A549; hASCs; fibrin mixed with type I collagen	Self‐assembled	^14^
MDA‐MB‐435S; B16‐F10	MDA‐MB‐435S; B16‐F10	Self‐assembled	^27^

Cell‐based 3D culture			
Cells for cancer research	Cells for organoids/3D tissue models	Vascularisation strategy	Ref
HSC‐3; SK‐Mel‐25; A2058; SCC‐9 culture with LN‐1 and LN‐2	HSC‐3	Self‐assembled	^58^
Panc1; PDAC; CSCs	Panc1; PDAC CSCs	Self‐assembled; Biological host	^59^
Highly malignant keratinocyte cell line HaCaT‐ras A‐5RT3	HaCaT‐ras A‐5RT3	Biological host	^60^
Caco‐2	Caco‐2; human ECs, PCs and FBs were embedded in a collagen (for in vivo implantation)	Self‐assembled; Biological host	^61^
KM12‐SM; HCT116	KM12‐SM; HCT116	Self‐assembled	^26^
H838; HCT 116	H838; HCT116	Self‐assembled	^13^
Human melanoma cells	ASCs; human melanoma cells	Self‐assembled	^62^
MCF10A; MDA‐MB‐231; A549; SW620	MCF10A; MDA‐MB‐231; A549; SW620; endothelial cells	Self‐assembled	^63^

Scaffold‐based 3D organoid culture			
Cell types	Cells for organoids/3D tissue models	Vascularisation strategy	Ref
MDA‐MB‐231(MM231); MCF7	MCF10A cells (normal‐like breast epithelial cell line); MCF7; MDA‐MB‐231 cells	Self‐assembled	^9^
HepG2	HepG2/HUVECs or HepG2/fibroblasts (L929)	Self‐assembled	^17^
MCF7	MCF10 (ATCC CRL‐10317); MCF7	Self‐assembled	^30^
MM231; T47D; BT47	MCF10A; MM231	Biological host	^16^
E‐98; E‐468	E‐98; E‐468	Biological host	^15^
CSC 248; CSC 974	CSC 248; CSC 974; hCMEC	Self‐assembled	^64^

Printed 3D organoids			
Cell types	Cells for organoids/3D tissue models	Vascularisation strategy	Ref
21PT; MDA‐MB‐231	Derived from breast cancer from C3(1)‐tag mice	Self‐assembled	^28^
Lung cancer cells derived from lung cancer patient	Lung cancer cells	Constructed artificial vascular channels	^29^
MDA‐MB‐231; MCF7; fragments from mice	MDA‐MB‐231; MCF7; Caco‐2 cells	Constructed artificial vascular channels	^65^
MDA‐MB‐231	MDA‐MB‐231; osteoblast	Constructed artificial vascular channels	^66^
MDA‐MB‐231; MCF7	MDA‐MB‐231; MCF7; human fetal osteoblast	Constructed artificial vascular channels	^67^
Diffuse‐type gastric cancer GA232; GA302a; GA352; GA356; GA052; GA265; GA121; GA302p	Diffuse‐type gastric cancer GA232; GA302a; GA352; GA356; GA052; GA265; GA121; GA302p	Constructed artificial vascular channels	^68^

CAM			
Cells for cancer research	Cells for organoids/3D tissue models	Vascularisation strategy	Ref
A549‐GFP; A549‐OKS	A549‐GFP; A549‐OKS	Growth from chicken embryo	^69^

*Note*: Categorised cancer cells and abbreviations in the table: Breast cancer cells: MDA‐MB‐231; MCF‐7; Hs‐578T; BCSCs (breast cancer stem cells); T47D; BT47; 21PT. Kidney cancer cells: 786‐O. Glioblastoma: GB3 GSCs; U87MG; E‐98; E‐468; CSC 248 and CSC 974 (patient‐derived glioblastoma multiforme stem cells). Squamous carcinoma cells: A431. Human hepatocellular carcinoma: HepG2. Colorectal adenocarcinoma cell: HCT116; SW 480; SW620; Caco‐2; KM12‐SM. Human ovarian cancer cell: SKOV3; OV90; OVCAR3. Human lung adenocarcinoma cell line: A549; H838. Oral tongue squamous cell: HSC (human oral tongue squamous cell carcinoma cell line); hSCC (human oral squamous cell carcinoma cell line). Pancreatic cancer cells: hPDAC (human pancreatic ductal adenocarcinoma); Panc1 (human pancreatic adenocarcinoma Parental cell). Gastric cancer cells: GA232; GA302a; GA352; GA356; GA052; GA265; GA121; GA302p. Melanoma cells: MDA‐MB‐435S; B16‐F10; SK‐Mel‐25; A2058. Other abbreviations: ECs (endothelial cells); HLECs (human lymphatic endothelial cells); iPSC‐ECs (induced pluripotent stem‐cell‐derived endothelial cells); hECs (human endothelial cells); HUVECs (umbilical vein endothelial cells); GFP (green fluorescent protein); NHLFs (normal human lung fibroblast); hCMEC (human brain endothelial cells); ASCs (adipose‐derived stem cells); hASCs (human adipose‐derived stem cells); CSCs (cancer stem cells); PCs (pericytes); FBs (fibroblasts).

**TABLE 3:** Summary of materials used for chamber/scaffold, ECM‐mimicking materials/scaffold, and vascular graft of selected studies.

Chamber/scaffold	ECM‐mimicking materials/scaffold	Vessel resources	Ref
PDMS	Collagen I and fibrinogen	HUVECs	^45^
PDMS	Matrigel	HUVECs	^46^
PDMS	Rat‐tail collagen type I and fibroblast	HLECs; HUVECs	^47^
PDMS	Rat‐tail collagen type I and fibrinogen, fibronectin	HLECs	^48^
PDMS	Rat tail collagen type I	HUVECs	^49^
PEEK	Fibrin and collagen I	Gelatin mix with ECs	^7^
PDMS	Gelatin; fibrin	HUVECs	^50^
PDMS	Gelatin; fibrin	HUVECs	^51^
PDMS	Lung FBs; collagen I	HUVECs	^52^
PDMS	Human dermal FBs; gelatin	HUVECs	^53^
PDMS	Rat tail collagen type I	HUVECs	^54^
PS	Fibrin gel and Matrigel	HUVECs	^55^
PDMS	AD; MC; fibrin	HUVECs	^56^
PDMS	NHLFs	HUVECs	^57^
PDMS	Fibrinogen	HUVECs with primary NHLFs	^14^
	Type I collagen and fibrin	hAMECs	
	Type I collagen and fibrin	RPEs with choroidal FBs	
PDMS	Fibrin gels	Human dermal microvascular lymphatic and blood ECs	^27^
	Myogel; Myogel‐LMA	HUVECs	^58^
	Matrigel; collagen I; mixture of Matrigel and collagen I		^59^
	Collagen I with and without NHDF		^60^
	Rat tail collagen type I; human fibroblasts‐derived collagen type I; human placenta collagen type I hydrogels; bovine collagen type I; human skin‐derived collagen type I	HECFCs	^61^
	Collagen type 1 sponges from pigs; fibrin gel	HUVECs with NHDF	^26^
	3D life hydrogel (produced by Cellendes) and RDG peptides	HUVECs with FBs	^13^
PDMS	PIC‐GRGDS peptides	HDMECs with FBs	^62^
	NHLF	HUVECs	^63^
RGD‐Alginate	RGD‐Alginate; fibroblasts; hMF	HOECs	^9^
PLGA	PLGA	HUVECs	
Gelatin	HDFs seeded on GPMs	HUVECs	^30^
TMS decellularised from mice breast tissue	Human GM637 or mouse NIH/3T3 fibroblasts; TMS		^16^
rBM	rBM		^15^
PLG	PLG	hCMECs	^64^
	Collagen–HA–pNIPAM; Matrigel; collagen; collagen plus fibrin	HUVECs and fibroblast	^28^
PEVA	LudECM; Matrigel; with or without iLFs	HUVECs	^29^
PVA; Silicone	Fibrin, gelatin, collagen, and Matrigel, human MSCs	HUVECs	^65^
	GelMA), PEGDA, HAP	HUVECs	^66^
	GelMA, PEGDA, HAP	HUVECs	^67^
PEVA	st‐dECM	HUVECs	^68^
	CAM	HUVECs with MSCs	^69^

Abbreviations: ADs (adipocytes); CAM (chorioallantoic membrane); GelMA (gelatin methacrylate); GPMs (gelatin porous microbeads); hAMECs (human adipose microvascular endothelial cells); HAP (hydroxyapatite nanoparticles).; HDFs (human dermal fibroblasts); HDMECs (human dermal microvascular endothelial cells); HECFCs (human endothelial colony forming cells); hECs (human endothelial cells); hMF (human mammary fibroblasts); HOECs (human outgrowth endothelial cells). Fibroblasts: FBs (fibroblasts); HUVECs (umbilical vein endothelial cells); iLFs (idiopathic pulmonary fibrosis ‐derived lung fibroblasts); MCs (mesothelial cells); MSCs (mesenchymal stem cells); NHDFs (normal human dermal fibroblast cells). Polymers: PDMS (polydimethylsiloxane); NHLFs (normal human lung fibroblasts); PEEK (polyetheretherketone); PEGDA (polyethylene glycol diacrylate); PEVA (poly (ethylene‐vinyl acetate)); PIC (polymer polyisociano peptide); PLG (poly(lac tide‐co‐glycolide); PS (polystyrene); PVA (polyvinyl alcohol). Other abbreviations: IPF (idiopathic pulmonary fibrosis); rBM (reconstituted basement membrane); RPEs (retinal pigment epithelial cells); st‐dECM (stomach‐derived decellularised ECM). The table: Endothelial cells: hCMECs (human brain endothelial cells); TMS (tissue matrix scaffold).

## 3 FABRICATION TECHNIQUES OF VASCULARISED ORGANOIDS

### 3.1 Methodologies for culture system setup

#### 3.1.1 Lab‐on‐a‐chip

**FIGURE 2:**
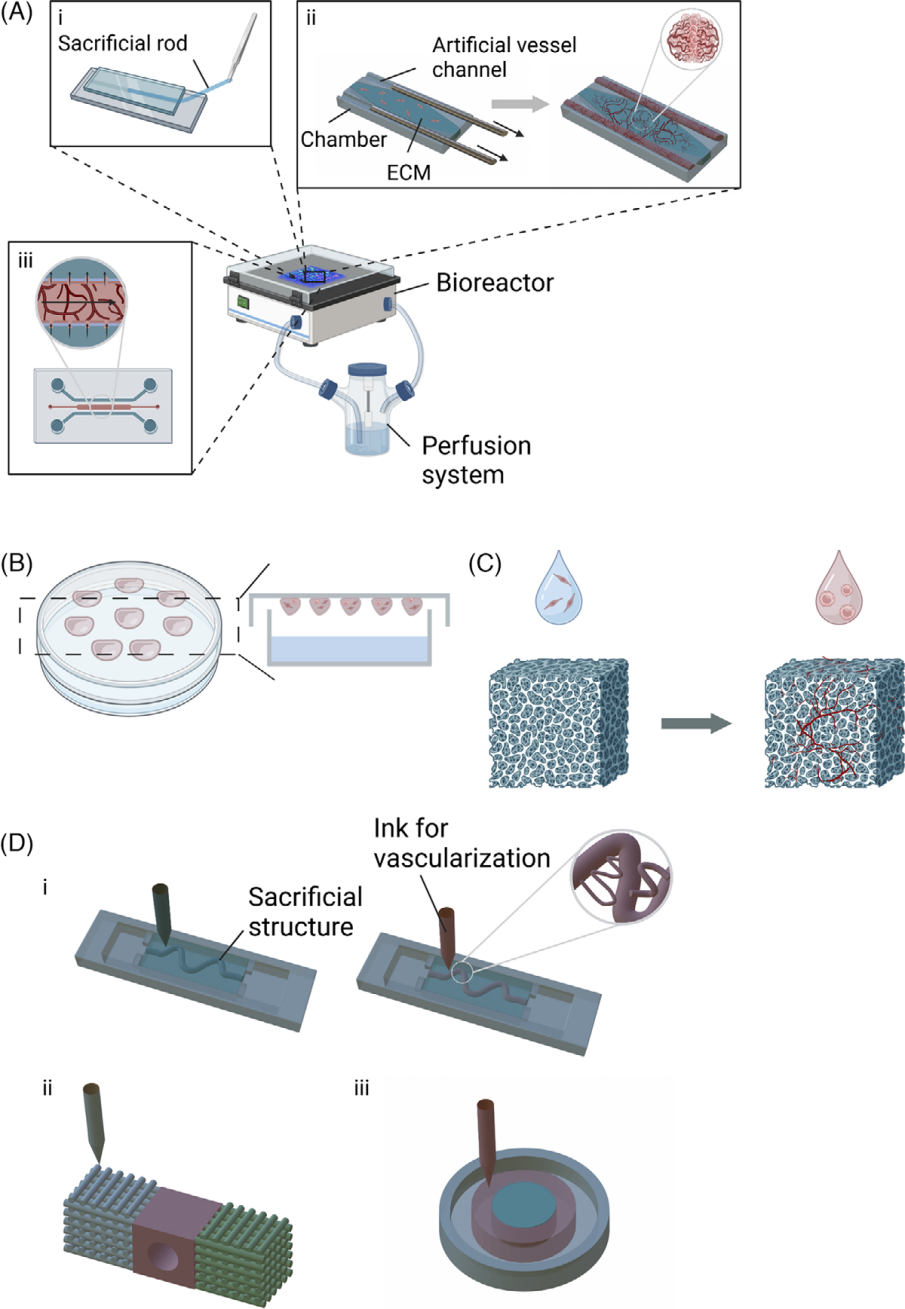
Schematic of vascularised organoid culture systems created by different techniques. (A) Classical Lab‐on‐a‐chip system: (i) Artificial vessel channel created within extracellular matrix (ECM)‐mimicking gel using a sacrificial rod.^49^ (ii) Device with a central cell culture chamber and two microchannels formed by needle removal. Endothelial cells seeded in side channels self‐assemble into a vascular network across both central and side regions.^14^ (iii) Chip with three channels: the central channel contains cells and ECM‐mimicking material, while side channels are filled with culture medium. Flow conditions are varied to study the effects of luminal and trans‐endothelial flow.^57^ (B) Hanging Drop Method: Vascularised organoids are cultured using biomaterial‐driven tissue engineering. (C) Pre‐vascularised scaffold: Organoids are grown on a scaffold that is pre‐vascularised to support further tissue development.^9^ (D) Bioprinting‐based systems: (i) Artificial vascular structures with customisable 3D geometries and channel diameters.^65^ (ii) Tumour‐vessel‐bone sandwich co‐culture model with central hole for an artificial vessel.^66^ (iii) Self‐assembled vascularised tumour model with a stromal region containing human umbilical vein endothelial cells and fibroblasts, and an inner region with breast cancer cells.^28^ Created with BioRender.com.

LoC devices, which integrate fluidics, biosensors, and optical systems to miniaturise laboratory functions onto a single chip, have emerged as a prominent technique for cultivating vascularised organoids.^70^ These LoC culture systems generally comprise bioreactors equipped with artificial ECM to supply the necessary space and nutrients for organoid development and vascularisation. Additionally, they incorporate a perfusion system that replicates the dynamic in vivo environment (Figure 2A).^45^, ^46^, ^47^, ^54^

Various strategies have been investigated to engineer vessel‐like structures within these systems. Several studies have successfully developed artificial vascular networks by creating channels where endothelial cells proliferate and form hollow lumens, emulating natural blood vessels. These channels are formed by injecting an ECM‐mimicking solution into microfluidic chambers containing removable polydimethylsiloxane (PDMS) rods (Figure 2A, i and ii). Upon removal of these rods, residual luminal structures are left behind. The PDMS rods can be customised in shape and diameter.^45^, ^47^, ^48^, ^49^, ^54^ This technique has been adapted for high‐throughput screening by incorporating iron particles into the PDMS rods.^49^ These flexible, metallic or magnetic rods can be removed automatically using magnets, facilitating simultaneous extraction of multiple rods.

Other fabrication techniques, including injection‐mouldeing,^46^ soft lithography,^56^, ^57^ and standard photolithography,^52^ have also been employed to create pre‐formed vascular channels on plastic substrates. Notably, the AngioChip platform,^50^, ^71^ which combines 3D stamping with LoC technology, has been utilised to produce biodegradable scaffolds with branched microchannel networks, enhancing vascularisation. This platform has been further developed into the InVADE system, which supports multiplexed perfusion and parallel screening of up to 20 3D tissues within a 96‐well plate format.^51^, ^53^ The system features a luminal scaffold with a 100 µm diameter and micro‐holes (15 µm diameter) representing vasculature, where endothelial cells form a confluent layer and sprout through the micro‐holes. The endothelialised lumen of the scaffold was visualised using CD31 immunostaining, which confirmed both the endothelial coverage and the formation of a confluent layer. The permeability of the endothelialised scaffold to large fluorescent molecules (70 kDa TRITC‐dextran) was comparable to the permeability of capillaries to proteins. The scaffold is surrounded by a parenchymal space that can be infused with ECM‐mimicking materials, such as fibrin gel, and populated with organ‐specific cells, allowing for the customisation of organ‐specific biological functions. Additionally, the system integrates cantilevers to measure tissue contraction.

In addition to pre‐existing vascular channels, several studies have shown the effective creation of self‐assembled vascular networks. For example, the U‐IMPCT platform employs mechanisms of spontaneous capillary flow and capillary burst valve mechanics to stimulate angiogenesis, tumour migration, and vascularisation without requiring external perfusion. This system, designed with a three‐channel architecture, promotes the development of microvascular networks through the establishment of concentration gradients and the adjustment of hydrodynamic conditions. They demonstrated the formation of a perfusable vascular network using a microbead assay. Microbeads with a diameter of 2  µm were observed to exclusively perfuse through the lumens of the vascular network, which had an approximate diameter of 30 µm.^55^

However, not all efforts have yielded complete vascular structures. Bonvin et al.^27^ developed a multi‐lumen fluidic system and successfully obtained multicellular structures with lumens. However, this study did not achieve the development of tubular vascular structures, as the focus was on the effect of blood flow stimulation on the morphogenesis of endothelial cell capillary formation, without further proceeding to a more macroscopic level of vascularisation. Importantly, this study shows that cells exposed to fluid flow underwent significant morphological changes, adopting a stellate shape. Conversely, cells maintained in a static environment did not display these alterations, underscoring the essential role of fluid flow in the process of vascularisation.^27^

#### 3.1.2 Cell‐based 3D culture

The most straightforward method for generating cell aggregates or basic organoids involves culturing cells on a hydrophilic surface.^26^, ^61^ The use of ultra‐low adhesion surfaces prevents cell attachment, while subsequent centrifugation promotes the formation of cell aggregates. Ehsan et al. directly co‐cultured tumour cells and endothelial cells within a cell suspension to develop the Prevascularised Tumour (PVT) model, which effectively promotes the formation of lumenised, vascularised networks of organoids within the surrounding matrix. ECs were labeled using CD31 antibodies. Fluorescent imaging of the vascular network revealed that the inner capillaries were shorter, more irregular in shape, and exhibited increased branching, in contrast to the radially sprouting capillaries on the periphery. The PVT model has been successfully adapted for various cancer cell lines, including MCF10A, MDA‐MB‐231, A549 and SW620.^63^ Another established technique is the hanging drop method (Figure 2B),^58^, ^59^ wherein cells aggregate at the bottom of a hanging drop. To enhance vascularisation within these organoids, the incorporation of biological hosts, such as mice or patient tissue slices, may be considered.^61^, ^62^ Optimal outcomes depend on the careful selection of ECM materials, as variations in ECM type – such as Matrigel or collagen I.^59^ – and differences in their concentration and source^61^ can significantly affect the development of organotypic structures. Additionally, proteins or vesicles such as MMPs^60^ and exosomes^26^ have been shown to influence the organoid microenvironment.

#### 3.1.3 Scaffold‐based 3D organoid culture

Scaffolds are essential for the development of vascularised organoids, providing a well‐structured network that supports cellular activities and facilitates tissue remodelling. These scaffolds mimic the natural ECM by offering both mechanical and biochemical cues necessary for tissue formation and integration with host tissues.^72^ To promote cell adhesion and vascular development, scaffolds must be porous (Figure 2C). Several techniques have been developed to create porous scaffolds. For instance, freeze‐drying combined with particle leaching uses RGD‐Alginate and sodium chloride (NaCl) particles, ranging from 150–250 µm in size, as porogens.^9^ Another method involves mixing scaffold materials, such as poly(lactic‐co‐glycolic acid) (PLGA), with NaCl particles (250–400 µm in diameter), compressing them into matrices, and then removing the NaCl with deionised water.^64^ Additionally, microfluidic technology can create porous scaffolds by dispersing gelatin, used as a porogen, in a PLGA solution, followed by gelatin removal.^17^, ^73^ Gelatin is able to act as both a porogen and a primary scaffold material. Porous scaffolds can also be generated by introducing toluene into a gelatin solution, cooling the mixture below 20°C, and removing the toluene through ethanol washing.^74^ These methods produce scaffolds that are not only porous but also biocompatible, making them suitable for 3D organoid culture.

In 3D culture systems, cells are either resuspended in a medium containing scaffolds that simulate the natural cellular environment^17^ or directly seeded onto the scaffold.^9^ However, research by Teixeira et al.^9^ has shown that various cell seeding methods, such as applying cell suspension to the top or both sides of the scaffold, do not significantly influence cell distribution.

Vascularisation of scaffold‐based 3D organoids can be achieved through several strategies. These include pre‐vascularising the scaffold with endothelial cells before introducing organ‐specific cells,^9^, ^30^ co‐culturing endothelial cells with organoid cells,^17^, ^64^ and implanting scaffolds into biological hosts.^15^, ^16^ Additionally, scaffold use has facilitated the cultivation of organotypic cells and the establishment of parenchymal zones, reflecting the intrinsic heterogeneity of tissues, such as breast tumours.^9^ For instance, Mazio et al.^30^ demonstrated that normal mammary epithelial cells (MCF10A) form acini‐like structures, while cancer cells create tumour‐like irregularities within a scaffold‐based 3D organoid culture, effectively replicating the in vivo heterogeneity of breast tumours.

#### 3.1.4 Bioprinting

Bioprinting, an advanced technique derived from additive manufacturing, enables the precise fabrication of biologically relevant materials and living cells into intricate 3D structures.^75^ This technology leverages computer‐aided design and the automated assembly of bioinks, followed by the cellularisation of the printed constructs.^76^, ^77^ Notably, six studies have utilised bioprinting to develop vascularised 3D organoids for cancer research, employing three distinct strategies for vascularisation.

One approach for constructing vascularised tissue models involves the use of pre‐formed hollow structures. For instance, Choi et al.^29^ advanced the development of a vascularised lung cancer organoid model through a multi‐step bioprinting process. They generated a hydrogel derived from the decellularised porcine lung extracellular matrix (LudECM) and utilised it as a bioink. The printed LudECM served as a scaffold for idiopathic pulmonary fibrosis‐derived lung fibroblasts (iLFs), effectively mimicking periparenchymal tissue. Additionally, LudECM was used to encapsulate lung cancer cells, forming lung cancer organoids (LCOs), which were subsequently printed within the LudECM/iLFs matrix. The formation of blood vessels was visualised using HUVECs labelled with red fluorescent protein. To enhance vascularisation, a microextrusion‐based technique was used during the bioprinting process, resulting in an endothelialised vessel embedded within the LudECM/iLFs/LCOs structure.

Kim et al.^68^ explored the interactions between printed gastric organoids and artificial vascular structures. They developed an artificial vascular channel within a stomach‐derived decellularised extracellular matrix (st‐dECM) by removing a gelatin rod and subsequently seeding the channel with human umbilical vein endothelial cells (HUVECs). A quantitative analysis was conducted using CD31 immunofluorescent staining of HUVECs to assess the area of sprouted vasculature and to measure VEGF secretion. The analysis revealed that lower VEGF secretion levels correlated with a lower sprouting tendency in organoids. Similarly, Hu et al.^65^ created a luminal structure by dissolving a preprinted polyvinyl alcohol (PVA) bioink embedded in a matrix composed of 7.5% porcine gelatin and 10 mg mL−1 fibrin (Figure 2D, i). PVA, chosen for its water solubility and excellent printability, served as a sacrificial scaffold to form artificial vascular channels.^65^, ^78^ This method successfully produced not only a primary vascularised channel but also smaller branching structures, demonstrating the precision of bioprinting in advanced tissue engineering. The formation of functional endothelial tissues was further validated through fluorescent imaging of mCherry‐labeled HUVECs and the diffusion of 70‐kDa dextran.^29^, ^65^, ^68^

An alternative approach involves the direct printing of chambers with integrated channels for endothelialisation, thereby bypassing the need for sacrificial structures. For instance, a tumour‐vessel‐bone co‐culture model was developed using gelatin modified with methacrylic anhydride and combined with polyethylene glycol (PEG) diacrylate (PEGDA) (Figure , ii). The printed vasculature was visualised by immunofluorescent labeling of ECs with vWF and CD31. Furthermore, endothelial cell proliferation was investigated, and CD31 gene expression was analysed via RT‐PCR to quantitatively assess angiogenesis.^66^, ^67^ Additionally, another study established an organotypic model without artificial channels, instead forming a stromal region with HUVECs and fibroblasts, and an inner region with breast cancer cells (21 PT). This setup led to the self‐assembly of capillary‐like structures (Figure 2D, iii). Total capillary tube length was measured after seven days of culture under normoxic and hypoxic conditions, with CD31 immunofluorescent staining of HUVECs. The results demonstrated that the capillary‐like structures formed under hypoxia exhibited significantly enhanced vessel formation, with vessels length twice as long as those observed in the normoxia control model. This finding mirrors the in vivo tumour environment and supports the use of this model for bioprinted lab‐on‐chip fabrication.^28^

In addition to the selected papers in this review, some recent studies further demonstrate the advantages of bioprinting. Pun et al.^79^ recently developed a PDMS‐free microfluidic platform to study the phenotypic heterogeneity and drug resistance of 3D bioprinted glioblastomas. They incorporated U87 cells from a human glioblastoma cell line and tissue‐specific endothelial cells (HBMECs, i.e. human brain microvascular endothelial cells) to create a vascularised microenvironment for the fabricated brain tumour model. Microphysiological systems (MPS) using vascularised organoids are exciting state‐of‐the‐art models that enable the study of tissues in dynamic 3D cultures, closely mimicking natural physiological conditions. A very recent study developed 3D bioprinted human vascularised liver organoids derived from iPSCs in a BME‐2 scaffold for high‐throughput screening and disease modelling, by co‐differentiation of mixed mesoderm‐derived vascular progenitor cells (VPCs) and EpCAM+ endodermal progenitor cells (EPCs).^80^ Wang et al. provide a dedicated step‐by‐step protocol that embeds bone marrow‐derived mesenchymal stem cells in a hydroxyapatite‐enriched hydrogel to fabricate large‐scale bone organoids capable of spontaneous mineralisation and vascularisation.^81^

#### 3.1.5 Chorioallantoic membrane culture

In addition to the aforementioned strategies, less conventional techniques have been employed to develop vascularised organoid culture systems for cancer research. The CAM assay is a notable method widely utilised in cancer research, angiogenesis studies, and drug testing.^82^ The CAM, a highly vascularised membrane present in bird embryos – especially in chicken eggs – functions as an interface for gas exchange during development.^83^ Miura et al.^69^ demonstrated the effectiveness of this technique by co‐culturing A549‐OKS cells, HUVECs, and mesenchymal stem cells, which led to the spontaneous formation of spherical organoid structures. Upon transplantation onto the CAM, these organoid spheres integrated with chick blood vessels, exhibiting vascularisation. The invasive behaviour of A549‐OKS cells further facilitated this process by twisting and infiltrating the host vasculature, enabling invasion into the CAM. The in vivo angiogenic activity was quantified using image processing software to calculate the area of new vessels and the mass area.

#### 3.1.6 Selection of a technique

In general, the selection of a technique depends on the specific research objectives and the available infrastructure. Table 4 compares the cost, operational complexity, model stability, and physiological relevance of various techniques, helping researchers identify the most suitable approach for their needs.

**TABLE 4:** Comparative evaluation of fabrication techniques.

Χ Disadvantage ● Neutral/Medium √ Advantage	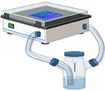	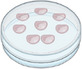	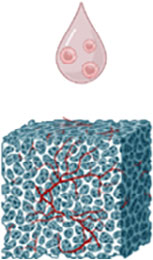	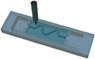	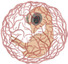
	LoC^14^, ^50^, ^51^	Cell‐based 3D culture^26^, ^59^, ^61^	Scaffold‐based 3D culture^16^, ^17^, ^30^	3D Bio‐printing^28^, ^29^, ^66^	CAM^69^
Cost	Χ	√	●	**Χ**	√
Operational complexity	**Χ**	√	●	**Χ**	●
Stability of the technique	●	√	●	●	●
Physiological relevance	√	**Χ**	●	√	●
Merits	High tunability Integration of various techniques	Straightforward Low cost Commercially available products	Better mechanical support Simple setup for material testing	High tunability Automation High precision	Low cost Naturally vascularised support
Drawbacks	Complex system setup Cost	Fragmentary vasculature Lacks dynamic fluidics	Lacks dynamic fluidics Limited tunability	Complex bioink design Requires specialised bioprinter equipment	Limited research applications Avian origin introduces variability

*Note*: Evaluation process: The symbols present the performance of each technique in corresponding aspects: **Χ** Disadvantage, ● Neutral/Medium, √ Advantage. The evaluation criteria for each parameter are as follows:

1.Cost: Includes human resources, devices and consumables.

2.Operation complexity: Assesses software requirements, specialised skills, hardware set up and maintenance.

3.Stability of the technique: Measures reproducibility, long‐term usability, operational sensitivity and precision.

4.Physiological relevance: Evaluates the extent to which the technique replicates the morphology and functions of native tissue,^84^ including functionality, homeostasis and structural morphology.

Partly created with BioRender.com.

Among these methods, the most widely used is the LoC technique, which integrates multiple approaches, including bioprinting,^7^ to create advanced culture systems. The scaffolds discussed here refer to prefabricated structures, such as chemically synthesised cubes or spheroids, produced without additive manufacturing. In contrast, bioprinting utilises specialised bioprinters, offering greater customisation in design and material distribution. However, this advantage comes with the added complexity of developing bioink and setting up printing systems. Developing bioinks poses significant challenges. These materials must exhibit low cytotoxicity, tolerate shear stress during and after the printing process, maintain optimal porosity, and possess excellent shear recovery properties.^66^, ^68^ Advances in biomaterials have shown promise in addressing these challenges. For example, silk fibroin (SF) can replicate the rheological behaviour of native silk gland proteins through methods such as adding organic solvents, pH adjustments, ultrasonication, chemical cross‐linking, and shear processing. These modifications allow SF to behave like a Newtonian fluid during extrusion through a bioprinter, followed by shear‐thickening to stabilise the structure after spinning.^85^

Cell‐based 3D culture is a more straightforward approach compared to LoC systems. However, 3D spheroid cultures and scaffold‐based approaches lack external perfusion systems, resulting in challenges such as limited vascularisation and restricted nutrient supply under static conditions. While some studies have reported the formation of vessel‐like structures in organoids, their vascular integrity and density are generally lower than those observed in LoC models. To address this limitation, some research groups have implanted these models into biological hosts to promote vascularisation and study interactions between organoids and in vivo tissues.^16^, ^59^, ^61^, ^62^ Another limitation of models without perfusion systems is their inability to replicate cancer cell invasion under dynamic fluid conditions. This limitation reduces their applicability in studying complex tumour microenvironments. Additional discussion on these challenges and potential solutions can be found in Supporting Information, section Extended Conclusion.

### 3.2 Material selection for vascularised organoid culture systems

The selection of materials is pivotal in the development of vascularised organoid culture systems. This section addresses four essential components: (1) chambers or scaffolds, which offer structural support for cell culture, fluid perfusion, and interactions between organoids and vascular networks; (2) ECM‐mimicking materials, which simulates the in vivo microenvironment of tissues or tumours to replicate biological activity; (3) endothelial cells for vascularisation, involving cells capable of forming or differentiating into vascular networks; and (4) additional molecular and cellular factors for vascularisation of organoids, crucial for promoting blood vessel formation and growth.

#### 3.2.1 Chambers and scaffolds

Chambers provide macro‐level mechanical support, essential for cell culture and the creation of fluid channels for perfusion. PDMS is the most widely used material (86%) for constructing bioreactors or chambers due to its cost‐effectiveness, high biocompatibility, and excellent gas permeability, which supports cell metabolism.^50^, ^86^ PDMS's versatility in manufacturing techniques, such as injection moulding and soft lithography, along with its self‐supporting structure and high elasticity,^87^ makes it an ideal choice for fabricating the base and sacrificial structures required for perfusable culture platforms.^49^

However, PDMS has a notable drawback: it tends to adsorb hydrophobic substances, including growth factors and small molecular drugs.^88^ This limitation has prompted the exploration of alternative materials such as polystyrene^50^ and polyether‐ether ketone,^7^ which are better at preserving cell‐cell signalling pathways and extending applications to pharmacokinetic studies. For bioprinted chambers, materials such as poly(ethylene‐co‐vinyl acetate) (PEVA)^29^, ^68^ and silicon^65^ have been utilised, with PEVA serving as a framework for bioinks and reinforcing the printed structures.^89^, ^90^

In the development of ECM‐mimicking scaffolds, various hydrogels such as alginate,^9^ gelatin,^30^ PLGA,^17^ and poly(lactide‐co‐glycolide) (PLG)^64^ are frequently employed. However, the potential adverse effects of degradation products and the inherent hydrophobicity of these polymers warrant further investigation. To address these challenges, Girdhari and Weimin^16^ developed a novel tissue matrix scaffold (TMS) characterised by 100 µm pores, created through the decellularisation and lyophilisation of mouse breast tissue. This TMS scaffold was seeded with MM231 cells and coated with TMS hydrogels to form a multilayered platform. In vivo studies demonstrated capillary formation as evidenced by immunofluorescence staining. However, vascularisation was not observed in vitro.

#### 3.2.2 ECM‐mimicking materials

The development of vascularised organoids, which emulate the architecture and functions of in vivo tissues and organs, is a pivotal aspect of tissue engineering. This multidisciplinary field, closely associated with advancements in biomaterials, including synthetic and modified natural materials, relies on the interaction between biomaterials and biological systems to create a supportive microenvironment for organoid vascularisation.^91^, ^92^ ECM‐mimicking materials, which replicate the functions of the ECM, play a crucial role in organoid growth and development. Consequently, significant research efforts have been dedicated to understanding the effects of these materials and to developing biomaterials tailored to specific biological needs.

Collagen, the most abundant protein in natural ECM,^93^ is the primary ECM‐mimicking material utilised in organoid culture for cancer research due to its profound influence on tumour growth. For example, breast tumour cell clusters preferentially orient along aligned collagen fibres.^30^ The source of collagen can also impact organoid growth significantly. Human bone marrow‐derived mesenchymal stem cells, for instance, display higher metabolic activity on collagen I from rat tails and human skin compared to other sources, such as bovine, human fibroblasts, or human placenta collagen. Collagen type I hydrogels derived from human skin exhibit properties, such as stiffness and resistance to enzymatic degradation, that are more comparable to xenogeneic‐derived collagen sources than other human‐derived collagens.^61^ Moreover, the density of collagen matrices affects cellular behaviour. Higher density collagen matrices have been shown to promote endothelial cell proliferation but may result in reduced overall cell attachment and coverage area, as evidenced by studies comparing low‐ and high‐density collagen I matrices.^48^ Additionally, researchers have explored innovative methods, such as combining collagen I with silk fibroin hydrogel using the ‘Freeform reversible embedding of suspended hydrogels (FRESH)’ technique, to balance ECM density with the demands of the manufacturing process.^28^

Matrigel, also known as basement membrane extract (BME), is widely regarded as the gold standard for tumour organoid culture due to its composition of key basement membrane proteins: laminin (∼60%), collagen IV (∼30%), entactin (∼8%), and the heparan sulphate proteoglycan perlecan (∼2–3%).^94^ This protein composition is essential for activating the autocrine/paracrine VEGF‐2 signalling pathway, thereby enhancing the vasculogenic phenotype.^59^ The proportion of Matrigel to collagen I within the ECM can significantly influence organoid morphology. For instance, pancreatic ductal adenocarcinoma cells (PDAC) exhibit loss of their organised structure when the ECM composition shifts from Matrigel to collagen I.^59^ Similarly, breast cancer cells (21PT) form acinar colonies in Matrigel, whereas collagen I fails to support this structure.^28^

Gelatin is another commonly used ECM material, providing initial support for cell adhesion, proliferation and ECM production.^30^ Its derivative, gelatin methacrylate, enhances mechanical strength.^66^ However, studies indicate that Matrigel may surpass gelatin in its ability to support breast cancer cell growth.^65^

Several studies have highlighted the advantages of using animal‐derived ECM materials due to their high biocompatibility and close resemblance to the in vivo architectural and chemical properties of natural tissues. One study introduced a TMS derived from mouse ECM, which was enriched in collagens, glycoproteins and glycosaminoglycans. Breast cancer cells cultured within this TMS exhibited scattering patterns akin to those observed in actual breast tumours, demonstrating a high degree of mechanistic mimicry of the in vivo physical environment. This TMS provided superior support for breast cell proliferation compared to synthetic polymer scaffolds, such as PLGA and polycaprolactone, as well as natural scaffolds including collagen and laminin‐rich ECM.^16^

Another example is LudECM, which includes collagen type VI, laminin 521, fibrinogen, and the basement membrane‐specific heparan sulphate proteoglycan core protein 2. LudECM has shown a more diverse matrisome protein profile compared to Matrigel, offering a more accurate approximation of the in vivo lung tissue microenvironment.^29^ Similarly, st‐dECM contains 21 stomach‐specific proteins and replicates matrix stiffness conducive to the regulation of proliferation via Yes‐associated protein (YAP) expression in tumours.^68^ These studies collectively underscore that tissue‐derived ECMs may provide a more comprehensive component profile and more accurately replicate the in vivo environment, potentially leading to improved outcomes in cancer research.

Advancements in the development of novel biomaterials are critical for the progress of physiologically functional organoids. For example, Salo et al.^58^ introduced Myogel, an ECM derived from human leiomyoma tissue, which, when combined with low‐melting agarose (LMA), demonstrated superior support for cancer cell invasion compared to Matrigel. The Myogel‐LMA mixture also enhanced capillary tube formation and improved cell viability.

Several selected studies have developed synthetic biomaterials for use in organoid culture systems, including PLGA,^17^ PLG,^64^ PEGDA.^66^, ^67^ Composite material consisting of collagen, hyaluronic acid, and poly(N‐isopropylacrylamide) (CH) has been shown to preserve a non‐invasive phenotype in breast cancer cells, closely mimicking primary tumour tissue.^28^ Despite their advantages, synthetic hydrogels often face challenges in biocompatibility due to the absence of integrin‐like cell binding sites. To address this, Zimoch et al.^62^ integrated the synthetic peptide Gly‐Arg‐Gly‐Asp‐Ser (GRGDS) into PIC peptides, thereby mimicking integrin binding sites and leveraging the thermosensitive properties of PIC peptides to enhance cell adhesion and simplify cell extraction. Kuehlbach et al.^13^ employed covalent peptide immobilisation on 3D Life hydrogels, which are enzymatically degradable by dextranase, to develop a dextran‐hydrogel‐based 3D tumour‐stroma model. This model allowed microtumour spheroids, cultured via the hanging drop method, to form tubule‐like structures that spread towards other spheroids. These hybrid hydrogels have shown the potential to support cell attachment and interaction in ECM‐mimicking environments.

In addition to synthetic materials used in the selected studies, several recent studies used suitable synthetic materials such as PEG,^95^, ^96^ methoxy‐PEG,^97^ polyacrylamide,^98^ PVA,^99^, ^100^ and polyisocyano peptides (PIC)^101^, ^102^, ^103^ for tissue and organoid fabrication. The landmark paper by Gjorevski et al.^95^ highlights the need for synthetic, animal‐free alternatives to Matrigel. Matrigel, derived from Engelbreth‐Holm sarcoma mice, is a natural biomaterial that provides a rich source of ECM components. Despite its advantages, the use of Matrigel is limited by its uncontrolled complexity, its batch‐to‐batch variability, its inability to adapt to different viscoelastic properties of individual tissues, and its potential for immunogen and pathogen transfer, making the search for alternatives essential. However, no fully defined biomaterial has yet been identified to replace Matrigel.

ECM‐like peptide hydrogels are another type of material combining both synthetic and natural aspects. Ultrashort self‐assembling peptide hydrogels have been successfully used for organoid fabrication and 3D bioprinting. These peptides are composed of no more than 3–7 natural amino acids.^104^ Peptide solutions containing lysine as a hydrophilic head group are excellent bioinks that allow immediate solidification of cell‐laden constructs under physiological salt conditions, avoiding harsh curing situations such as UV exposure, stressful chemical additives and prolonged temperature conditions.^105^, ^106^

#### 3.2.3 Endothelial cells for vascularisation of organoids

**FIGURE 3:**
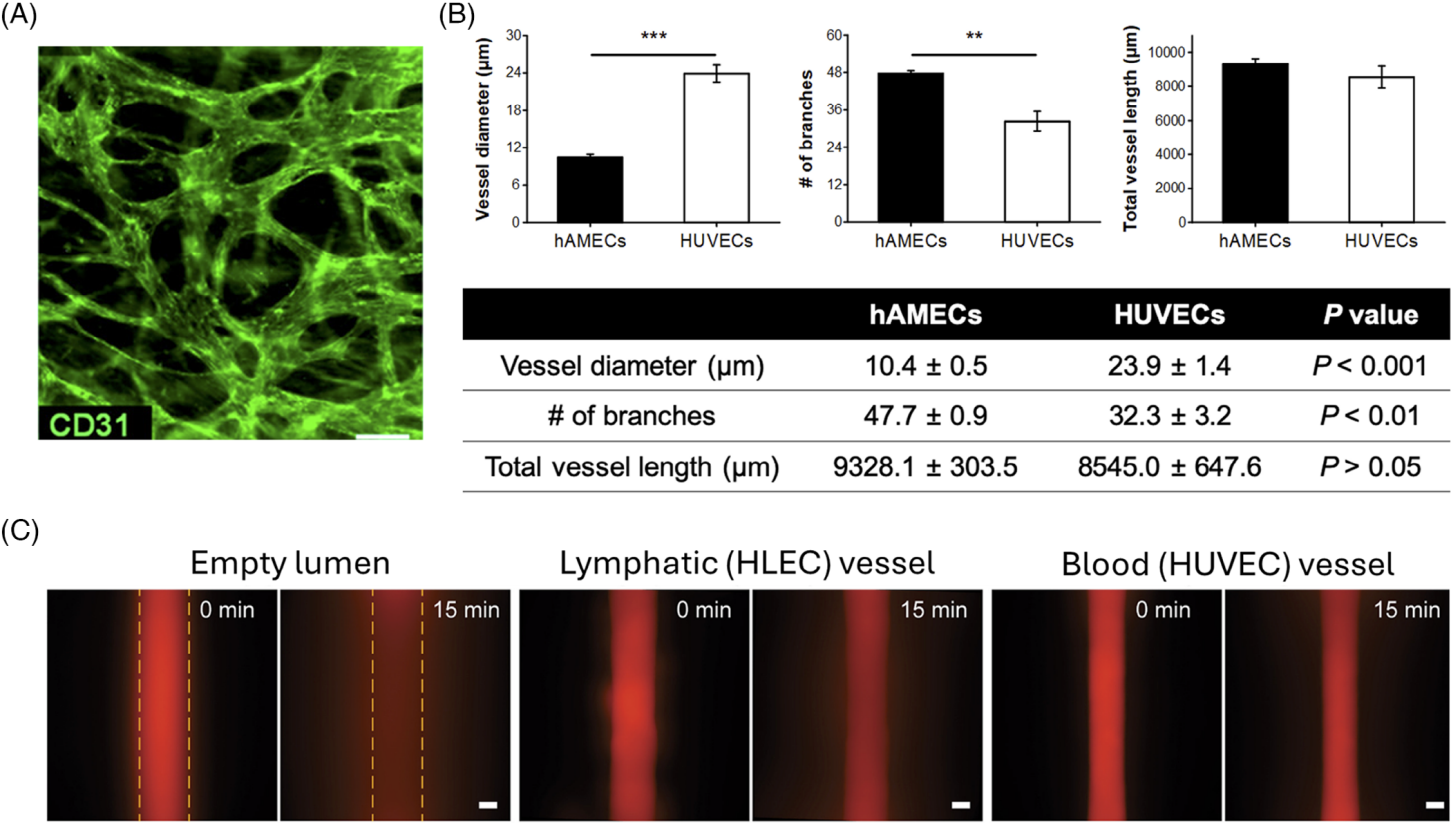
Morphological features of blood vessels assembled by human umbilical vein endothelial cells (HUVECs), human adipose microvascular endothelial cells (hAMECs), and human lymphatic endothelial cells (HLECs). (A) The vessels derived from hAMEC have smaller diameters, but they are more branching than the HUVEC‐derived vessels. No statistical difference was found in the total length of the two types of vessels. (B) Immunofluroesence staining of CD31 expressed by the HUVEC‐derived vessels in the self‐assembled vascular network. Scale bar, 100 µm. (C) Diffusion profile of 70  kDa dextran diffusion in empty, lymphatic, and blood vessels over 15 min. HLEC vessels were leakier than HUVEC vessels. Dashed lines indicate lumen boundaries. Parts (A) and (B) were reprinted with permission from Ref. (14). Copyright 2019, American Chemical Society. Part (C) was reprinted with permission from Ref. (47). 2019 Elsevier Ltd. All rights reserved.

In organoid research, the choice of endothelial cells for vascularisation is crucial, as the cellular origin profoundly impacts vessel morphology, functionality, permeability, and overall model success. In 25 of 37 studies, HUVECs (Figure 3A) were utilised to construct artificial vessels.

HUVECs, primary endothelial cells derived from the umbilical vein, are renowned for their exceptional ability to survive up to five months in in vitro culture systems.^107^ Their strong expression of essential endothelial markers and signalling molecules related to vascular homeostasis, such as endothelial nitric oxide synthase (eNOS) and nitric oxide, makes them the most commonly used cell type for vessel formation in 3D models.^13^, ^108^, ^109^

However, HUVECs are not the only option for organoids vascularisation, and the morphology, functionality, and permeability of the resulting vessels can vary significantly depending on the source of endothelial cell. For example, blood vessels formed from primary human adipose microvascular endothelial cells are smaller but exhibit greater organisation and density compared to those derived from HUVECs (Figure 3B).^14^ Similarly, vessels formed by human lymphatic endothelial cells show 1.3‐fold higher permeability than those formed by HUVECs when exposed to interstitial flow of a 70 kDa dextran solution (Figure 3C).^47^ These variations highlight the importance of carefully selecting the appropriate endothelial cell type for vascularisation, particularly when developing the parenchymal compartment of organoids. Other endothelial cell types used in various studies include human dermal microvascular lymphatic and blood endothelial cells,^27^ human endothelial colony forming cells,^61^ human outgrowth endothelial cells,^9^ and human brain endothelial cells.^9^ Nevertheless, direct comparisons between these cell types and HUVECs under identical culture conditions are limited, restricting a comparative evaluation of their relative impacts on vascularisation.

HUVECs are widely used in tissue engineering due to their robust capacity for capillary morphogenesis. Despite their advantages, they have notable limitations. Their venous origin is sometimes considered as a disadvantage, though developmental studies suggest that arterial endothelial cells can arise from venous precursors. Additionally, HUVECs are derived from allogeneic tissue, and their proliferation potential is inherently limited.^110^

The vascularisation of organoids represents a crucial step for mimicking in vivo environments and ensuring organoid viability and functionality. Several well‐established protocols exist to achieve this:

(1) Co‐culture with ECs: A common approach involves the co‐culture of tumour cells with ECs in an organoid‐forming environment, which facilitates the self‐assembly of vascularised organoids. The addition of supportive stromal cells further stabilises the vascular structures. The process is driven by key elements, including angiogenic growth factors like VEGF (vascular endothelial growth factor), bFGF (basic fibroblast growth factor), and angiopoietins, in combination with ECM components such as collagen or Matrigel, which provide a supportive 3D scaffold.^111^, ^112^

(2) Growth factor supplementation: This strategy focuses on the supplementation of the culture medium with pro‐angiogenic growth factors to stimulate vascularisation. The addition of VEGF, often in combination with fibroblast growth factor (FGF) and platelet‐derived growth factor (PDGF), has been demonstrated to facilitate endothelial cell differentiation and vessel sprouting. The concentrations and timing of growth factor addition need to be optimised for specific organoid types in order to achieve efficient vascularisation.

(3) Self‐organising approach: This method leverages the intrinsic potential of organoids to form vasculature. By incorporating angiogenic lineage induction pathways, organoids can spontaneously develop vascular structures. Supporting cells facilitate the stabilisation of these vessels, while endogenous VEGF expression and matrix remodelling contribute to the efficacy of this technique.^111^

#### 3.2.4 Additional molecular and cellular factors for vascularisation of organoid

In addition to endothelial cells, critical molecules such as fibrin and growth factors play a fundamental role in angiogenesis by facilitating the migration and proliferation, and therefore the stabilisation of new blood vessels. Other cell types, such as fibroblasts, are also vital in this process, as they secrete ECM components and paracrine signals that support vessel maturation and enhance the structural integrity of the vascular network.^13^

Among growth factors, VEGF is the most crucial and specific for vessel formation,^113^ with its levels increasing as vascularised organoids are cultured.^13^ Jiménez‐Torres et al.^49^ demonstrated that a VEGF gradient could drive vessel sprouting toward regions of higher VEGF concentration. VEGF primarily acts through the vascular endothelial growth factor receptor (VEGFR), with different receptor subsets influencing various aspects of the vascularisation process.^114^ For example, VEGFR‐2 expression is associated with the formation of angiogenic mimicry.^59^ VEGF specifically binds to fibrinogen and fibrin with high affinity. Fibrin, an insoluble fibrous protein formed from fibrinogen through enzymatic reactions with thrombin, creates a gel that supports vessel formation.^26^ Fibroblasts are significant sources of VEGF, thereby stimulating endothelial cell sprouting and growth, as well as influencing extracellular ECM composition and remodelling.^9^, ^55^ Properly oriented gradients of pro‐angiogenic factors from fibroblasts enhance vascular induction.^52^ Additionally, fibroblasts produce matrix MMPs, which degrade collagen in organoids, promote tumour invasion, and support the formation of new, immature vessels.^60^ The elongated morphology of fibroblasts often indicates a conducive environment for organoid vascularisation.^62^ Lai Benjamin et al.^53^ showed that co‐culturing fibroblasts with organoids significantly increased organoid size, gel compaction and collagen deposition compared to organoid monocultures. However, fibroblasts also limited the diffusion of small molecules, such as carboxyfluorescein diacetate, within the system.

Beyond VEGF, other factors also impact vascularisation. For instance, hydroxyapatite nanoparticles have been shown to accelerate HUVEC migration and vessel formation.^66^ Additionally, Woenne et al.^60^ found that overexpression of 2,3,7,8‐tetrachlorodibenzo‐p‐dioxin‐inducible poly (ADP‐ribose) polymerase (TIPARP) increased vessel area and mass. TIPARP influences ECM remodelling pathways, modulates MMP7 levels, and inhibits pro‐inflammatory cytokines to promote angiogenesis.

## 4 APPLICATIONS OF VASCULARISED ORGANOIDS IN CANCER RESEARCH

Following an overview of the methodologies and materials used to create vascularised organoids, this section examines their potential applications and highlights key findings from selected studies.

### 4.1 Cross‐talk between angiogenesis and tumour cells

**FIGURE 4:**
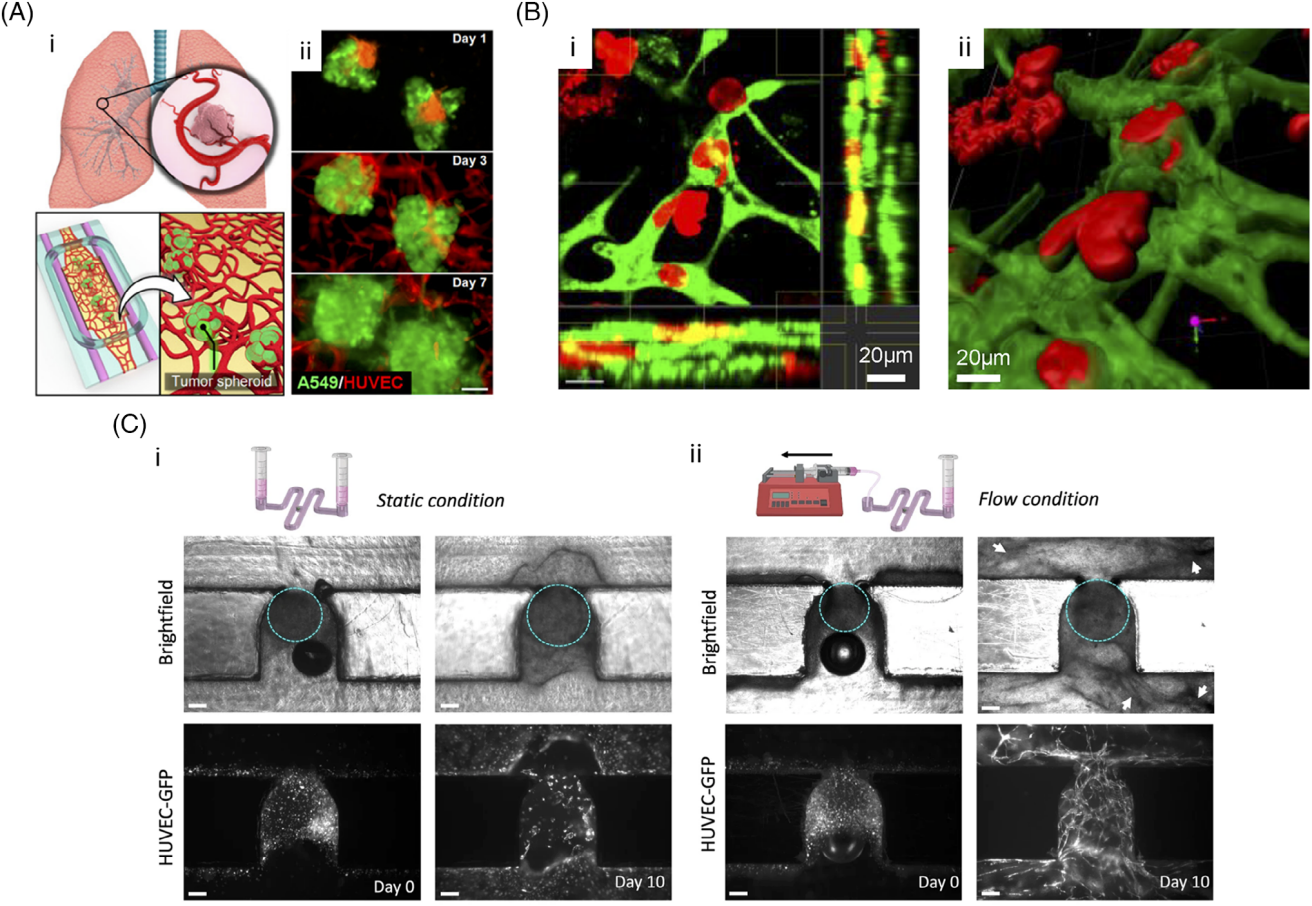
Vascularised organoids models. (A) Self‐assembled perfusable microvasculature with A549 organoids: (i) Lab‐on‐a‐chip mimicking malignant lung tumours. (ii) A549 cells (green) integrate with red fluorescent protein – human umbilical vein endothelial cells (HUVECs) (red) vasculature from day 1 to day 7. Scale bar, 50 µm. (B) 3D evaluation of the colorectal cancer vascular invasion model: (i) Horizontal section image (ii) 3D reconstructed image with Imaris software. (C) Blood vessel organoids (bright‐field, dotted cyan line) and green fluorescent protein – HUVEC network development on‐chip (day 0–10): (i) Static and (ii) flow conditions. Day 10 bright‐field images reveal extracellular matrix remodelling enhanced under flow conditions. The white arrows highlight the presence of black structures surrounding the trap site, indicative of active cell processes. Parts (A) was reprinted with permission from Ref. (14). Copyright 2019, American Chemical Society. Parts (B) was reprinted with permission from Ref. (26). Part(C) was reprinted with permission from Ref. (115).

Vascularised organoids provide a valuable in vitro platform for examining the complex interactions between tumour cells or organoids and angiogenesis, closely replicating in vivo processes that are otherwise challenging to observe and quantify. The relationship between tumour architecture and vascular development is both dynamic and reciprocal. Blood vessels within tumour organoids establish critical channels for gas and nutrient exchange, which has been shown to lead to increased cell proliferation and enhanced cancer cell migration compared to non‐vascularised models.^29^, ^67^ Advanced imaging techniques such as confocal microscopy^26^ (Figure 4B) and green fluorescent protein imaging^51^ reveal that cancer cells actively invade and migrate along vessels, whereas fibroblasts do not display similar invasive behaviours. Tumour cells are capable of both migrating along vessels (intravasation) and traversing them (extravasation).^66^ Additionally, Yanagisawa and colleagues^26^ reported a decrease in E‐cadherin expression in cancer cells within capillaries compared to those outside, suggesting that epithelial‐to‐mesenchymal transition – a crucial step in tumour invasion – occurs within these vascular structures.

The presence of tumour cells within a vascularised organoid significantly enhances the vascular network, resulting in increased volume coverage, branch density and vessel diameter.^30^, ^69^ Tumour‐induced angiogenesis, especially within distances of less than 50 µm, demonstrates more efficient sprouting behaviour.^68^ However, tumours also promote excessive branching of immature tubules and the formation of poorly lumenised vessels.^52^, ^63^ Additionally, exosomes released by cancer cells have been shown to diminish tight junctions in the vascular endothelium, thereby increasing vascular permeability.^26^

### 4.2 Drug responsiveness in tumour organoids

Vascularised organoids have emerged as valuable tools for evaluating drug efficacy and therapeutic responses in cancer research. These models have effectively demonstrated the therapeutic potential of various drugs, such as Cisplatin^13^ and Bevacizumab,^69^ and have provided critical insights into tumour responses to different treatments. For instance, Paek et al.^14^ (Figure 2A, ii) utilised vascularised organoids to validate the targeting efficacy of antibody‐functionalised liposomal nanocarriers directed at the vascular endothelium activated by intercellular adhesion molecule 1, even under conditions of spatially graded inflammation (Figure 4A). Wong et al.^54^ delivered doxorubicin and Doxil at clinically relevant concentrations to visualise and quantify the chemotherapeutic responses of tumour and endothelial cells, demonstrating how vascularised organoids contribute to guiding clinical dosing regimens and improving new drug delivery systems.

Drug responses in 3D models often differ from those observed in 2D models, with 3D systems generally offering a closer approximation to clinical outcomes. For example, although Tamoxifen and Paclitaxel inhibit breast cancer cell proliferation in both 2D and 3D models, the inhibition is less pronounced in the 3D context.^16^, ^66^ Wang et al.^17^ attributed this discrepancy to the presence of tight junctions within multicellular aggregates, which delay drug internalisation. The impact of vascularisation on drug delivery efficiency is multifaceted; while vessels facilitate drug entry into tumours, they can also impede efficacy due to endothelial barriers. In 2D and less vascularised 3D models, drugs often accumulate on the outer surface.^17^ However, when Paclitaxel was perfused through vascularised organoids, cytotoxicity was predominantly observed near the spheroid centers.^14^

Drug resistance in these models is influenced not only by the denser 3D structure but also by tumour fibrosis. For example, the IC50 value for Poziotinib – a drug typically considered sensitive – was approximately 370 times higher in fibrotic liver cancer organoids compared to non‐fibrotic counterparts.^29^ The presence of vasculature further complicates drug efficacy due to endothelial barriers. The diffusion of dextran was significantly impaired when endothelial cells and fibroblasts were co‐cultured, compared to when they were cultured individually. Additionally, following treatment with 0.2µM Poziotinib, the number of remaining proliferating cells was notably higher in vascularised liver cancer organoids co‐cultured with activated liver fibroblasts compared to non‐vascularised models.^29^ Similarly, Lai et al.^53^ observed that vascularisation limited the cytotoxic effect of Gemcitabine on PDAC organoids within a dynamic perfusion system, with this limitation being more pronounced in the presence of fibroblasts.

The integration of multiple organ models in vitro provides a valuable approach to studying the interactions between normal tissues and cancer organoids. For instance, enhanced tumour toxicity was observed when a liver model was coupled with a breast cancer model and perfused with the chemotherapeutic agent Tegafur.^51^ Additionally, patient‐derived organoids offer predictive insights into individual chemotherapy responses, enabling personalised treatment strategies. For example, organoids derived from gastric cancer patients have demonstrated varied sensitivities to 5‐fluorouracil and Oxaliplatin.^68^ Furthermore, vascularised organoids are emerging as promising tools for high‐throughput screening, facilitating the efficient testing of therapeutic agents across multiple disease models. Lai et al.^50^ developed the InVADE platform, a 96‐well system integrating 32 perfusable vascularised tissues, underscoring the potential of these models for advancing drug discovery and personalised medicine.

### 4.3 Elucidation of cytokine dynamics in vascularised organoids

Recent studies have expanded our understanding of how vascular networks influence tumour growth beyond merely facilitating nutrient and gas transport to include the regulation of cytokine secretion. Cytokines are low‐molecular‐weight proteins that play crucial roles in cellular communication, differentiation, proliferation and immune responses.^116^ Vascularised organoids offer a practical platform for examining the impact of cytokines on vessel formation and organoid growth. For example, vascularised organoid models have shown increased levels of the key angiogenic factor VEGF over time.^13^ Additionally, heightened collagen I density in the ECM has been associated with elevated concentrations of the pro‐inflammatory cytokine IL‐6, which leads to increased vascular permeability. Notably, IL‐6 levels were found to rise when tumour cells were co‐cultured with vascular structures.^48^

Further research by Infanger et al.^64^ revealed that cancer stem cells (CSCs) within a vascularised environment upregulated the IL‐8 receptors CXCR1 and CXCR2, resulting in enhanced CSC growth and migration. Gong et al.^47^ compared cytokine profiles in the lymphatic vessel system co‐cultured with breast cancer‐associated fibroblasts (CAFs) versus normal fibroblasts. Their findings demonstrated that CAFs significantly increased the secretion of pro‐tumourigenic growth factors such as granulocyte‐colony stimulating factor and hepatocyte growth factor by 8‐fold and 15‐fold, respectively, and pro‐inflammatory mediators including IL‐6 and IL‐8 by 20‐fold and 15‐fold, respectively.

## 5 ETHICAL AND REGULATORY ASPECTS OF VASCULARISED ORGANOIDS TECHNOLOGY

The development of vascularised tumour models using human cells and tissues raises significant ethical considerations that require careful attention. These cells are typically obtained from donors via biopsies or derived using pluripotent stem cell techniques. One key ethical issue is the limitation of informed consent, particularly when donors may not fully understand the potential downstream applications of their cells in advanced research and technology. To address this, clear and transparent communication about the intended research, potential applications, and data protection is essential. The use of vascularised organoids is regulated by national laws and international guidelines. However, these regulations vary significantly across countries leading to potential ethical grey areas. Moreover, as technology advances, existing regulations may need to be updated to address emerging issues and ensure responsible use. The establishment of formalised guidelines that align with regulatory standards is crucial to supporting the ethical development and application of these models.^117^, ^118^

## 6 CONCLUSIONS

Vascularised organoids represent a major innovation in the development of physiologically relevant 3D models, more closely replicating the metabolic processes, microenvironments and cellular interactions observed in vivo. These organoids are increasingly recognised as superior to traditional 2D and animal‐based models due to their enhanced biological and physical relevance. This review provides a detailed overview of the techniques and materials used in the generation of vascularised organoids, alongside an exploration of their various applications.

Identifying a universally applicable technique for generating vascularised organoids across all cell types remains challenging. However, the significance of dynamic fluidic systems in enhancing vascularisation is increasingly recognised. Although not always mandatory, research demonstrates that dynamic fluid systems significantly improve the replication of in vivo vascular environments. Fluid flow plays a crucial role in organ morphogenesis, homeostasis and pathogenesis by providing mechanical stimulation to cells, transporting nutrients and signalling molecules, and modulating the ECM.

Mechanical stimuli from fluid flow include shear stress (tangential forces parallel to the cell surface along the flow direction) and pressure stress (perpendicular forces normal to the cell surface). These forces regulate essential cellular processes such as proliferation, differentiation, quiescence and migration. In vascularisation, shear stress is pivotal for endothelial cell alignment, lumen formation and vessel stabilisation, while pressure stress influences vascular permeability and integrity. Microfluidic systems enable precise control of these forces, replicating in vivo‐like conditions that promote functional vasculature formation within organoids.^119^ For example, Caco‐2 organoids develop 3D protrusion‐like structures under perfusion, a feature absent in static cultures. Similarly, tumour fragments cultured under dynamic conditions exhibit higher viability compared to non‐perfused systems.^65^ Endothelial cells show greater alignment with fluid flow under dynamic conditions compared to static environments.^47^ The integration of fluid flow into organoid culture systems enhances vascular maturation, improves nutrient delivery, waste removal, and paracrine signalling. Quintard et al.^115^ demonstrated that vascularised organoids cultured under flow conditions exhibit mature endothelial networks, enhanced ECM remodelling, higher growth rates, and RNA profiles closely resembling those of in vivo transplanted organoids (Figure 4C). These advancements are critical for the viability and functionality of vascularised organoids.

Vascularised organoids have shown great promise as in vitro models for drug efficacy testing, offering significant advantages over animal models and traditional 2D cell cultures. For example, PDAC CSCs in 3D models exhibited higher growth rates compared to parental cells, consistent with findings from nude mouse experiments,^59^ whereas 2D models showed differing trends.^120^ Organoids derived from genetically diverse backgrounds can predict individualised drug responses, while vascularisation ensures more accurate distribution of drugs, immune cells and cytokines within organoids, mimicking the body's natural responses.^121^ For instance, paracrine factors secreted by 3D organoids were found to more effectively increase neurosphere size and CSCs DNA content compared to 2D cultures.^64^ Similarly, organoids demonstrated stronger correlations with patient data in gene expression studies for targeted therapies, such as gastric cancer response to Ramucirumab.^68^ Notably, tumour organoids co‐cultured with endothelial cells exhibited substantial vascularisation after implantation in mice, where capillary structures anastomosed with host vasculature with minimal foreign body reactions.^61^, ^64^ These findings highlight the clinical potential of vascularised organoids in cancer research, offering insights into tumour biology and advancing therapeutic evaluations.

To achieve clinical implementation of precision oncology, standardising and improving the reproducibility of organoid generation is essential. Multi‐region tissue sampling can more accurately model intratumour heterogeneity,^122^ while advancements in microfabrication techniques could standardise organoid derivation^123^ and enable high‐throughput drug screening.^124^

However, replicating the in vivo cancer microenvironment remains a challenge due to the complex biological and mechanical properties involved. Although engineered or self‐assembled vascular structures have shown promise, achieving fully functional and hierarchical vascular networks remains a significant goal. Endothelial cells have been observed to form lumen structures as early as the second day of culture under appropriate environments.^52^ While some studies have maintained organoid cultures for up to 28 days,^58^ many are limited to 1 week,^27^, ^46^, ^55^ which constrains the development of biologically relevant processes.

Future efforts should focus on the development of long‐term vascular networks with arteries, veins and capillaries.^125^ Strategies such as co‐culture, co‐differentiation and organoid assembly could facilitate the establishment of long‐lasting, perfusable vascular systems.^126^ Alternatively, integrating vascularised models with biological hosts, such as mice^127^ or CAM,^69^ or advanced perfusion systems may enable the creation of networks that more closely mimic in vivo conditions.

Recent advances in induced pluripotent stem cell (iPSC) technology have enabled the differentiation of iPSCs into endothelial cells (iPSC‐ECs) capable of forming vessel‐like networks in vitro and in vivo. These networks exhibit features of mature capillaries, including collagen IV, laminin and hollow lumens, although they show reduced sprouting activity compared to HUVECs. Despite these differences, iPSC‐ECs hold promise for the generation of vascularised organoids with perfusable networks and a more mature endothelial phenotype.^110^, ^128^

Studies highlight the crucial role of vascular networks in tumour progression, emphasising the therapeutic potential of anti‐angiogenic strategies in cancer treatment. Vascularised models provide a valuable platform for assessing the efficacy of novel therapeutic agents or approaches targeting angiogenesis, with the potential to induce tumour dormancy.^129^ Furthermore, genetic engineering holds significant promise for advancing vascularised organoid development through targeted gene modifications.^130^ For example, Palikuqi et al.^131^ demonstrated that transient reactivation of mature human endothelial cells via transduction with the ETS variant transcription factor 2 can generate tubulogenic and perfusable endothelial cells. Additionally, pathogenic genes and mutations can be directly studied in organoids using gene knockout or conditional gene deletion techniques,^132^ with CRISPR screening serving as a powerful tool to elucidate the tumour‐suppressive functions of specific biomolecules.^133^

In summary, vascularised organoids represent an innovative tool in cancer research, unlocking new possibilities for constructing 3D organotypic tissues with integrated vascular networks that closely mimic the in vivo tumour microenvironment. These models surpass traditional methods in their physiological relevance, offering unprecedented insights into tumour growth, proliferation mechanisms, and innovative treatment strategies. This review highlights the cutting‐edge techniques and materials driving the development of these sophisticated in vitro models, while also exploring their vast potential across cancer research. By analysing the key factors influencing the success of these systems, this review equips researchers with a deeper, more refined understanding of the current landscape, setting the stage for the creation of even more effective and clinically relevant vascularised organoid models. As this field advances, the promise of accelerating discoveries in cancer biology and therapy becomes more tangible, sparking optimism for transformative breakthroughs in the treatment of cancer.

## AUTHOR CONTRIBUTIONS

RZ: Conceptualisation, methodology, visualisation, writing – original draft preparation, reviewing and editing. DB: Validation, reviewing and editing. JF: Visualisation, reviewing and editing. AL: Reviewing and editing. SL: Reviewing and editing. CAEH: Methodology, supervision, reviewing and editing. CB: Conceptualisation, methodology, supervision, project administration, writing – original draft preparation, reviewing and editing.

## CONFLICT OF INTEREST STATEMENT

The authors declare that they have no competing interests

## ETHICS APPROVAL AND CONSENT TO PARTICIPATE

Not applicable.

## Supporting information



Supporting information

Supporting information

## Data Availability

Data sharing is not applicable to this article as no new data were created or analyzed in this study.
