# Supplementary figures and images for: Vascularised organoids: Recent advances and applications in cancer research
Source: Clin Transl Med. 2025 Mar 5;15(3):e70258. doi: 10.1002/ctm2.70258 (PMC11882480; doi:10.1002/ctm2.70258)

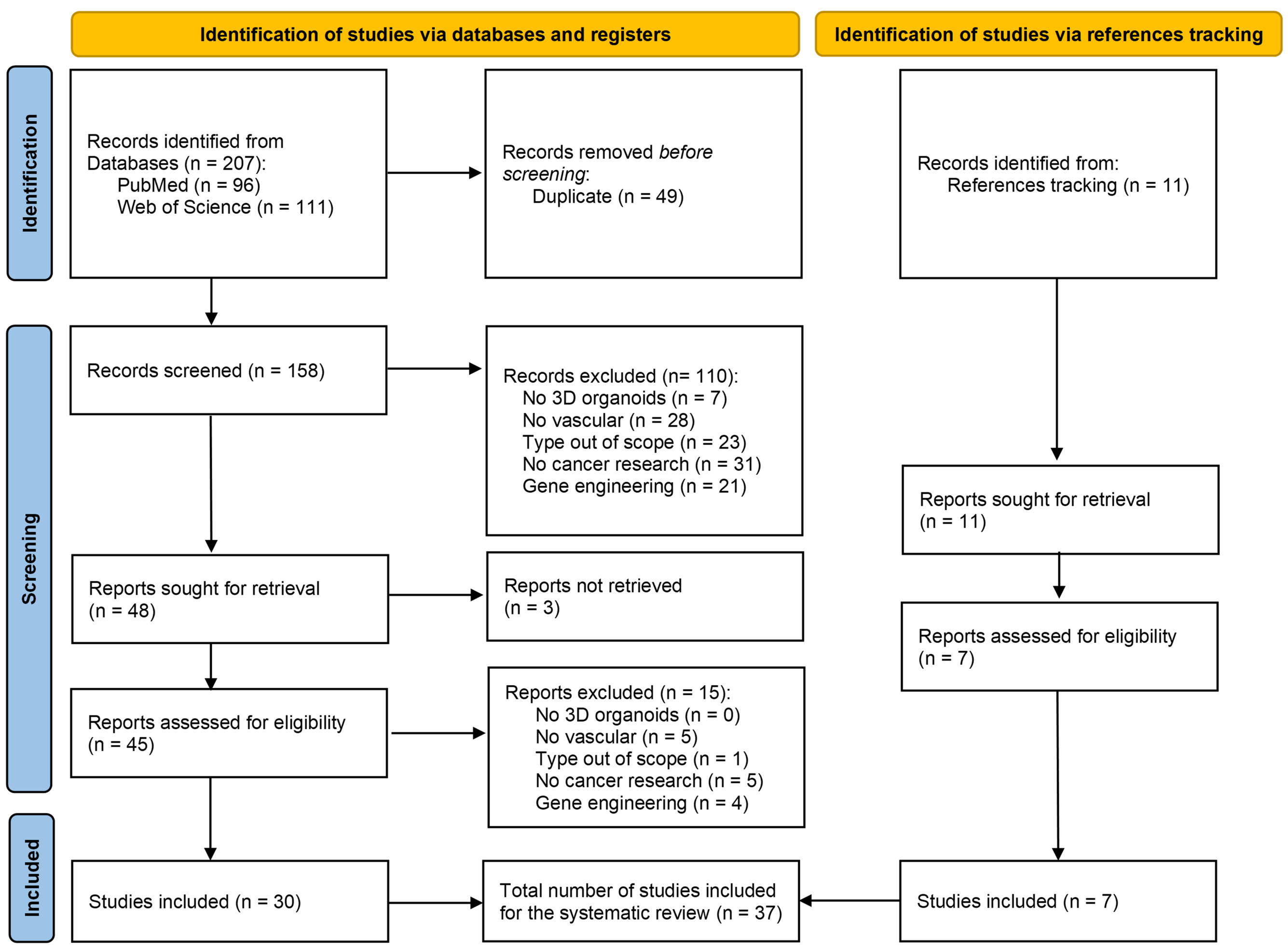

Supplement: Supplementary file 1 — Supporting information [file CTM2-15-e70258-s001.png]
