# Supplementary material for: Vascularised organoids: Recent advances and applications in cancer research
Source: Clin Transl Med. 2025 Mar 5;15(3):e70258. doi: 10.1002/ctm2.70258 (PMC11882480; doi:10.1002/ctm2.70258)
Supplement: Supplementary file 2 — Supporting information [file CTM2-15-e70258-s002.docx]

**Literature selection**

***Literature search strategy***

To identify studies focused on the development of vascularized organoids for tumour research, a comprehensive systematic search was performed using the PubMed and Web of Science databases. The search string was designed by combining key terms as follows: (hydrogel OR biomaterial OR Matrigel OR polymer* OR collagen OR fibrin OR gelatin OR alginate OR HA) AND (organoid* OR “3D multicellular tissue” OR “3D tissue” OR organotypic) AND (vascular* OR vessels) AND (tumour OR cancer).

In addition to database searches, manual cross-referencing, and complementary reviews were conducted to include relevant articles not captured in the initial search. The search targeted full-text articles published in English between 2008 and 2023, focusing on 39 selected papers that met the inclusion criteria. The search was conducted on May 3^rd^, 2024.

***Study selection criteria***

Articles were selected based on the following inclusion criteria: (1) original research articles, (2) studies involving 3D multicellular tissue or organoid systems, and (3) research specifically addressing organoid vascularisation in the context of cancer. Exclusion criteria included: (1) duplicate publications, (2) books, review articles, conference proceedings, notes, and in silico studies, (3) studies not focused on the development or application of 3D multicellular tissues or organoids, (4) studies not investigating vascularisation processes, (5) research unrelated to cancer, (6) studies primarily centred on gene engineering, and (7) articles where full-text access was unavailable.

***Data extraction***

The results from the database searches were exported into an MS Excel spreadsheet, where duplicates were automatically identified and removed. Titles and abstracts were then critically evaluated by two independent reviewers (RZ and CB), who assessed each study against the established eligibility criteria to identify potentially relevant articles. For studies where the abstracts lacked sufficient detail, a full-text review was conducted. The final selection of articles was determined through discussions between the two reviewers. The selection process is illustrated in the PRISMA flow diagram (Figure S1).

Qualitative data from the selected studies were extracted based on the predefined criteria. Table 1 provides a summary of key data, including:

(1) Technique: Identified based on the terminology used in the study or by assessment of the authors when the techniques were not clearly defined;

(2) Cell types used in cancer research;

(3) Cell types used in organoid or 3D tissue models;

(4) Vascularisation strategy: Studies were categorized as self-assembly, artificial vascular channel construction, or biological host models based on the culture system design and study outcomes.

Table 2 summarizes the materials used, including:

(1) Chamber/scaffold materials;

(2) Extracellular matrix (ECM) materials;

(3) Vascularisation materials.

It is noted that each study may employ multiple materials within each category. Additionally, information on the application of vascularized organoids in the selected studies was extracted for further analysis.


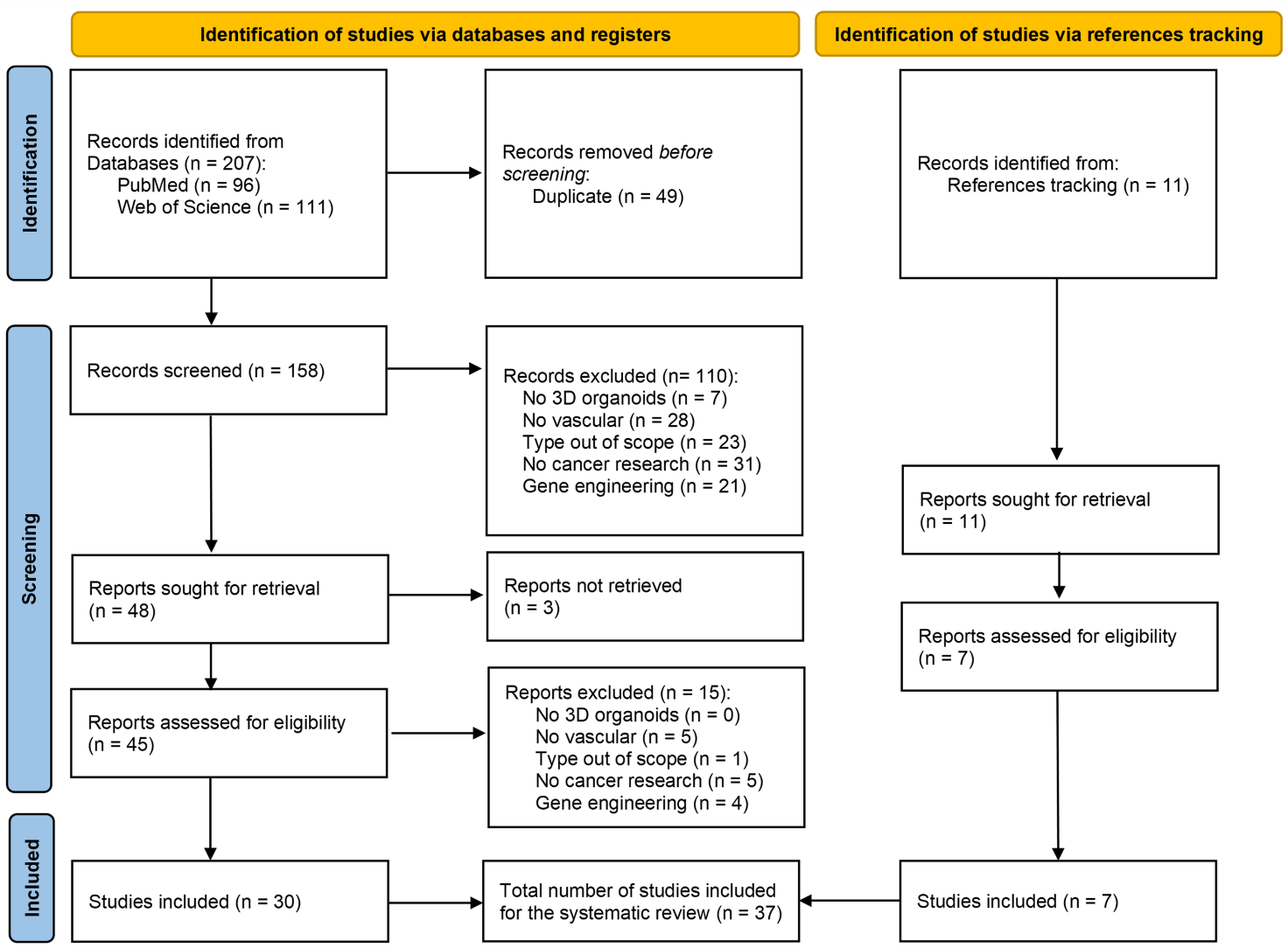


**Figure S1:** PRISMA flowchart for article selection. Template adapted from *The PRISMA 2020 statement: an updated guideline for reporting systematic reviews* ^44^

**Extended Conclusion**

The incorporation of cellular and other biological molecules can significantly impact the morphology and functionality of vascular networks and organoids. For instance, the presence of adipocytes in a model results in narrower and more branched vessels compared to models with endothelial cells alone. Mesothelial cells can extend branch length and reduce the number of branches per lateral area, but this effect is observed only in the presence of adipocytes^56^. Additionally, cells are commonly cultured in Dulbecco’s Modified Eagle Medium (DMEM) before being transferred to ECM materials or scaffolds, with DMEM being a widely used medium for spheroid culture^52,71^, particularly in hanging drop culture methods^15,134^.

The formation of blood vessels begins with the proliferation and growth of endothelial cells. While human umbilical vein endothelial cells (HUVECs) are the most frequently employed, other endothelial cell types, such as human endothelial colony-forming cells (HECFCs) and outgrowth endothelial cells (OECs)—subpopulations within endothelial progenitor cells—show great promise due to their robust proliferation rates and potent neovascularisation capabilities^135,136^. Additionally, fibroblasts, fibrin, and fibrinogen are often incorporated into culture systems, as these components play crucial roles not only in vascularisation but also in ECM formation^137^. Fibroblasts are key in the development and maintenance of anatomically distinct ECM-rich connective tissues through the deposition and remodelling of ECM components. They promote the aggregation of endothelial cells into capillary-like networks and can sustain these networks *in vitro* for up to 21 days^138^.

Fibrin, derived from the biological conversion of fibrinogen, serves as a substrate for epithelial cell migration and is integral to processes such as hemostasis and wound healing^139–141^. Fibrin also plays a role in cancer cell proliferation and angiogenesis by binding growth factors such as VEGF and fibroblast growth factor (FGF)^142^. Furthermore, fibrin influences cellular behaviour, including alignment, angiogenesis, and migration, by modulating fibre alignment and stiffness gradients^143^. VEGF is a critical regulator of angiogenesis, driving endothelial cell proliferation, migration, new blood vessel formation, and endothelial cell survival^144^. Although these biomolecules and materials are not always indispensable for the development of vascularized organoid culture systems, a deeper understanding of their roles could significantly enhance future research and applications in this field.

The establishment of an optimal microenvironment for organoid vascularisation requires the careful selection of techniques and materials, as environmental conditions can profoundly influence angiogenesis and organoid development. For instance, hypoxic conditions have been shown to significantly enhance the formation of capillary-like networks by HUVECs compared to normoxic conditions^28^. Additionally, hypoxia has been demonstrated to promote tumour cell migration, leading to larger migration areas and greater migration distances^63^. The use of 96-well transwell culture plates for extended culture durations (exceeding 10 days) has been found to reduce the damage associated with insufficient oxygen and nutrient diffusion between upper and lower cell layers^13^.

This review provides a comprehensive overview of the applications of vascularized organoids in cancer research, highlighting their ability to model the complex interactions between artificial vascular networks and tumour growth. Vascularized organoids reveal a reciprocal relationship between vascular networks and tumour proliferation: tumours stimulate increased blood vessel size and branching, while vascular networks facilitate cancer cell migration and growth by supplying nutrients and creating migration channels. Cytokine studies offer deeper insights into the interactions between tumours, blood vessels, and the microenvironment.

Studies about the application of vascularized organoids demonstrate the feasibility of using vascularized organoids as *in vitro* models for drug efficacy testing, offering advantages over animal models and traditional 2D cell cultures. For example, PDAC CSCs (pancreatic ductal adenocarcinoma cancer stem cells) in 3D models exhibited elevated growth rates compared to parental cells, consistent with results obtained in nude mouse experiments^59^, while a different trend was observed in 2D models^120^. Moreover, paracrine factors secreted by 3D organoids were found to more effectively increase the size of neurospheres and the DNA content of CSCs compared to 2D cultures ^64^. When examining gene expression responses to targeted therapies, such as gastric cancer response to Ramucirumab, organoids demonstrated a stronger correlation with patient data than 2D models^68^. Notably, tumour organoids co-cultured with endothelial cells showed substantial vascularisation following implantation in mice, with capillary structures anastomosing with the host vasculature, accompanied by minimal foreign body reactions^61,64^. These findings underscore the significant clinical potential of vascularized organoids in cancer research, particularly in advancing understanding of tumour biology and enhancing the evaluation of therapeutic interventions.

Replicating the *in vivo* cancer microenvironment poses a significant challenge due to the intricate biological and mechanical properties involved. Although various studies have successfully engineered artificial and self-assembled vessels, fully replicating the complexity of *in vivo* vascular networks remains a difficult goal. Endothelial cells have been observed to form lumen structures as early as the second day of culture in appropriate environments^52^. Some studies have sustained organoid cultures for up to 28 days^58^; however, many are limited to a single week^27,46,55^, which restricts the development of biologically relevant processes. Future research should prioritize the development of long-term vascular networks with hierarchical structures^125^, including arteries, veins, and capillaries. Integration of vascularized models with biological hosts, such as mice^127^ or CAM^69^, or the design of advanced perfusion systems, may facilitate the formation of vascular networks that more closely mimic *in vivo* conditions.

Studies also highlight the role of vascular networks in tumour progression, emphasizing the potential of anti-angiogenic therapies for cancer treatment. Vascularized models offer a valuable platform for assessing the efficacy of novel therapeutic agents or strategies aimed at inhibiting angiogenesis, potentially leading to tumour dormancy^129^. Moreover, genetic engineering holds significant promise for advancing vascularized organoid development through targeted gene modifications^130^. For instance, Palikuqi et al.^131^ demonstrated that transient reactivation of mature human endothelial cells via transduction with the ETS variant transcription factor 2 (ETV2) could generate tubulogenic and perfusable endothelial cells. Additionally, pathogenic genes and mutations can be evaluated directly in organoids through gene knockout or conditional gene deletion^132^, with CRISPR screening being a useful tool for elucidating tumour-suppressive effects of specific biomolecules^133^.

To facilitate the clinical implementation of precision oncology, enhancing the standardization and reproducibility of organoids is critical. Multi-region tissue sampling can more accurately model intratumor heterogeneity, aiding in the collection of representative tumour tissues^122^. Furthermore, advances in microfabrication could standardize organoid derivation^123^ and enable high-throughput drug screening^124^, reducing the variability associated with differing protocols.

Despite the complexity of the *in vivo* environment, which presents challenges for *in vitro* models, rapid advancements in biomaterials, micromanufacturing, and computational tools are making the future of these models increasingly promising. Vascularized organoids hold great potential as powerful tools for studying disease mechanisms, optimizing cancer therapies, advancing regenerative medicine, and ultimately, reducing the reliance on animal testing.

**References in Supporting Information**

134. Korff T, Augustin HG. Integration of Endothelial Cells in Multicellular Spheroids Prevents Apoptosis and Induces Differentiation. *J Cell Biol*. 1998;143(5):1341-1352. doi:10.1083/jcb.143.5.1341

135. Baiguera S, Ribatti D. Endothelialization approaches for viable engineered tissues. *Angiogenesis*. 2013;16(1):1-14. doi:10.1007/s10456-012-9307-8

136. Fuchs S, Ghanaati S, Orth C, et al. Contribution of outgrowth endothelial cells from human peripheral blood on in vivo vascularisation of bone tissue engineered constructs based on starch polycaprolactone scaffolds. *Biomaterials*. 2009;30(4):526-534. doi:10.1016/j.biomaterials.2008.09.058

137. Plikus MV, Wang X, Sinha S, et al. Fibroblasts: origins, definitions, and functions in health and disease. *Cell*. 2021;184(15):3852-3872. doi:10.1016/j.cell.2021.06.024

138. Costa-Almeida R, Gomez-Lazaro M, Ramalho C, Granja PL, Soares R, Guerreiro SG. Fibroblast-Endothelial Partners for Vascularisation Strategies in Tissue Engineering. *Tissue Eng Part A*. 2015;21(5-6):1055-1065. doi:10.1089/ten.tea.2014.0443

139. Wolberg AS. Fibrinogen and fibrin: synthesis, structure, and function in health and disease. *J Thromb Haemost*. 2023;21(11):3005-3015. doi:10.1016/j.jtha.2023.08.014

140. Weisel JW, Litvinov RI. Fibrin Formation, Structure and Properties. In: Parry DAD, Squire JM, eds. *Fibrous Proteins: Structures and Mechanisms*. Springer International Publishing; 2017:405-456. doi:10.1007/978-3-319-49674-0_13

141. Pereira M, Rybarczyk BJ, Odrljin TM, Hocking DC, Sottile J, Simpson-Haidaris PJ. The incorporation of fibrinogen into extracellular matrix is dependent on active assembly of a fibronectin matrix. *J Cell Sci*. 2002;115(3):609-617. doi:10.1242/jcs.115.3.609

142. Vilar R, Fish RJ, Casini A, Neerman-Arbez M. Fibrin(ogen) in human disease: both friend and foe. *Haematologica*. 2020;105(2):284-296. doi:10.3324/haematol.2019.236901

143. Ceccarelli J, Putnam AJ. Sculpting the blank slate: how fibrin’s support of vascularisation can inspire biomaterial design. *Acta Biomater*. 2014;10(4):1515-1523. doi:10.1016/j.actbio.2013.07.043

144. Melincovici CS, Boşca AB, Şuşman S, et al. Vascular endothelial growth factor (VEGF) - key factor in normal and pathological angiogenesis. Rom J Morphol Embryol. 2018;59(2):455-467.
